# Methylene Blue Attenuates 3-Nitropropionic Acid-Induced Oxidative Stress and Mitochondrial Dysfunction in Striatal Cells: Therapeutic Implications in Huntington’s Disease Neuropathology

**DOI:** 10.3390/ijms262110672

**Published:** 2025-11-01

**Authors:** Hannah K. Hale, Kayla M. Elias, Shawn Ho, Gunnar F. Kwakye

**Affiliations:** Neuroscience Department, Oberlin College, 119 Woodland Street, Oberlin, OH 44074, USA; hhale@oberlin.edu (H.K.H.); kelias2@oberlin.edu (K.M.E.); sho@oberlin.edu (S.H.)

**Keywords:** Huntington’s disease, methylene blue, 3-nitropropionic acid (3-NPA), oxidative stress, mitochondrial dysfunction

## Abstract

There are no disease-modifying treatments available for Huntington’s disease (HD), a neurodegenerative disease caused by a genetic mutation in the Huntingtin gene. Previous research suggests that disruptions in the bioenergetics of the mitochondria and increased oxidative stress are potential inducers of HD. Therapies that enhance antioxidant pathways intend to target and attenuate the overproduction of reactive oxygen species associated with mitochondrial dysfunction. We have investigated the effect of Methylene Blue (MB) as a potential therapy for HD. MB is a small molecule demonstrated to exhibit neuroprotective effects in other neurodegenerative disease models, including Parkinson’s and Alzheimer’s, by attenuating the oxidative stress pathways implicated in their pathophysiology. We used an established striatal cell model of HD expressing wild-type (ST*Hdh*^Q7/Q7^) or mutant (ST*Hdh*^Q111/Q111^) HTT and a chemical inducer of HD, 3-Nitropropionic acid (3-NPA), to determine the HD-specific mechanisms regulated by 3 h of MB pre-treatment. Upon 24 h of exposure to 3-NPA, mutant HD cells exhibited a significant concentration-dependent decrease in cell survival and a concomitant increase in cell death compared to wild-type, confirming that 3-NPA exacerbates mutant HTT neurotoxicity. Examination of mitochondrial membrane potential and mitochondrial function in the striatal cells by JC-1 and ATP assays, respectively, revealed MB mediated neuroprotection against 3-NPA-induced reduction in mitochondrial activity. Immunoblotting analysis revealed that MB restores baseline expression of oxidative-stress-related proteins, including HO1 and p62, in both wild-type and mutant cells exposed to 3-NPA. Our findings establish a novel neuroprotective role of MB in both genetic and pharmacological models of HD, suggesting that MB might be a promising therapeutic candidate for altering the underlying pathophysiology of HD by improving mitochondrial function.

## 1. Introduction

HD is a debilitating inherited neurodegenerative disease that primarily targets the medium spiny neurons of the striatum and other parts of the brain [1], resulting in a triad of symptoms, namely motor, psychiatric, and cognitive. The mean age of onset of HD is 40 years, with early symptoms manifesting as involuntary movements (chorea), hyperkinesia, and difficulties with executive functions [2]. The irreversible course of progression lasts 15 years on average, leading to life-altering disability, reduced quality of life, and, ultimately, death [1]. HD has an autosomal dominant inheritance pattern caused by a mutation in the Huntingtin gene (*HTT*) found on chromosome 4 [3]. The mutated *HTT* sequence features an elongated series of CAG repeats, resulting in a polyglutamine expansion that disrupts the normal folding and function of the HTT protein [2,3,4]. HTT is ubiquitously expressed and involved in various physiological functions in the body, including nuclear import, protein-protein complex formation through axon trafficking, and transcriptional regulation of neuronal survival, which are critical for striatal neuron activity and survival [5]. A pathophysiological hallmark of HD is the accumulation of the mutated HTT protein, which results in several pathogenetic mechanisms, including altered mitochondrial dynamics, excitotoxicity, and dysregulated transcription of proteins necessary for the normal functions of the neuron [5,6].

In neurodegenerative diseases, including HD, there exists substantial evidence that oxidative stress is a critical contributor to pathology [7]. Endogenous antioxidant defense, modulated by the nuclear factor E2-related factor (NRF2)/antioxidant response element (ARE) signaling mechanism, plays a crucial role in protecting cells from oxidative stress by regulating the expression of genes involved in antioxidant defense and detoxification. Evidence shows that the NRF2/ARE transcriptional activity is decreased in a striatal cell model of HD [8,9] and cells expressing mutant HTT [10].

Mitochondria are critical organelles that support the physiological processes of the cell through ATP production, regulation of intracellular calcium, alteration of the cell’s redox potential, scavenging of free radicals, and regulation of part of the proteasome system [11]. Oxidative phosphorylation occurs at the electron transport chain (ETC), a series of protein complexes embedded in the inner mitochondrial membrane that couple electron and proton transfer through redox reactions to drive the production of ATP [12]. In the striatum of patients with HD, the expression of subunits of complex II is reduced, causing a decrease in complex activity [13]. Defects to mitochondrial complex II induced by mHTT have also been demonstrated in in vitro and in vivo models [11,14]. Other transcriptional changes include alterations to the Peroxisome proliferator-activated receptor gamma coactivator (PGC1-α), a master regulator of mitochondrial function [15]. Decreased expression of PGC1-α mRNA in post-mortem brain samples of HD patients, as well as in striatal and animal models of HD, has been implicated in contributing to mitochondrial dysfunction and the progression of striatal neuron degeneration [15,16,17,18]. Fission, the process by which a mitochondrion separates into two, and fusion, in which two mitochondria merge, are key processes for regulating mitochondrial dynamics [8,19]. In striatal neurons expressing mHTT, there is increased mitochondrial count and fragmentation due to the fission process [20,21]. Levels of mRNA and expression of proteins associated with fission and fusion, such as Dynamin-related protein (DRP1 and), fission 1 protein (Fis1), dominant optic atrophy 1 (OPA1), and Mitofusions 1 and 2 (MFN1 and MFN2), are altered in neurons expressing mHTT [9,20,22,23]. Specifically, an increase in DRP1 and Fis1, and a decrease in fusion proteins, has been reported in the striatum and cortex of post-mortem brains from HD patients and in cells expressing mHTT [20,24]. In a striatal cell model of HD, greater susceptibility for cell death due to increased DRP1 and mitochondrial fragmentation was observed compared to WT and was corrected by treatment with pro-fusion proteins [25].

Cell death mediated by impairment in mitochondrial dynamics following metal exposure has been reported in striatal cells expressing mHTT, indicating a relationship between genetic and xenobiotic alterations of mitochondria [26]. 3-Nitropropionic acid (3-NPA) is a mycotoxin and irreversible chemical inhibitor of mitochondrial complex II that mimics the pathology observed in HD patients, animals, and cell models by inducing GABAergic medium spiny neuron degeneration in the striatum. 3-NPA increases oxidative stress in the cell and causes secondary excitotoxicity due to increased sensitivity to glutamate levels and dysfunctional energy production, ultimately leading to cell death [27,28]. In vivo, administration of 3-NPA induces proliferative changes in the dendrites of striatal spiny neurons, similar to those observed in HD [27]. Previous studies have reported motor and behavioral impairments, including abnormal gait and increased anxiety in animal models treated with 3-NPA. Therefore, it is used as an established *HD* pharmacological model in vitro and in vivo [27,28,29].

Therapeutic approaches that mitigate or impede neuronal dysfunction and loss in neurodegenerative diseases are necessary and timely. Methylene Blue (MB) is an organic, heterocyclic, thiazine dye originally employed in the textile industry. Its ability to cross the blood–brain barrier and high affinity for mitochondria make it potentially well-suited for targeting disorders associated with mitochondrial dysfunction [30,31]. At the mechanistic level, MB interacts with electron carriers (such as NADH in the mitochondria) and is reduced to leucomethylene blue (LMB), allowing it to “shuttle electrons” across the electron transport chain [30,32]. This unique redox property enables it to cycle between oxidized and reduced forms, facilitating ATP production as an alternate electron carrier and attenuating inhibitors to the electron transport chain. Ultimately, this reduces excessive reactive oxygen species and preserves mitochondrial health [30,33,34,35]. MB has been utilized as a therapeutic agent for septic shock and methemoglobinemia and has recently been studied in the context of lessening oxidative stress associated with neurodegenerative diseases, including Alzheimer’s Disease (AD) and Parkinson’s [33,36,37,38]. Previous work established the influence of MB on neural damage induced by the neurotoxin rotenone in vivo. It revealed that MB could attenuate oxidative stress associated with inhibited cytochrome oxidase and prevent behavioral impairment triggered by rotenone [39]. In the context of HD, MB has therapeutic promise in in vivo and in vitro models [40]. In HD, MB treatment prevented insoluble and soluble mHTT aggregates in cortical neurons and reduced the behavioral phenotype of HD in mice [40]. Importantly, MB, an FDA-approved drug, is well-tolerated at lower doses; only at high concentrations of 100 µM does it exhibit genotoxic effects in isolated mitochondria from the brain [35,41]. In addition to altering the expression of antioxidants, MB may preserve the function of other mitochondrial proteins by protecting the organelle from oxidative damage [42]. The reported beneficial effects of MB on neurodegeneration support the necessity to investigate the effects of this drug in HD. To our knowledge, MB has not been investigated in genetic in vitro striatal models of HD with an overlaid 3-NPA pharmacological model to mimic gene-environment interaction. The study aims to investigate the potential effects of MB against mutant HTT and 3-NPA-induced neurotoxicity and neurodegeneration in an established striatal cell model of HD. We hypothesize that MB will combat oxidative stress and improve mitochondrial health, augmenting neuroprotection against genetic and chemically induced HD phenotypes in an established striatal cell model.

## 2. Results

### 2.1. Establishing Mb and 3NPA Dose–Response Effects on Cell Viability in Wild-Type and Mutant HD Cells

**Figure 1:**
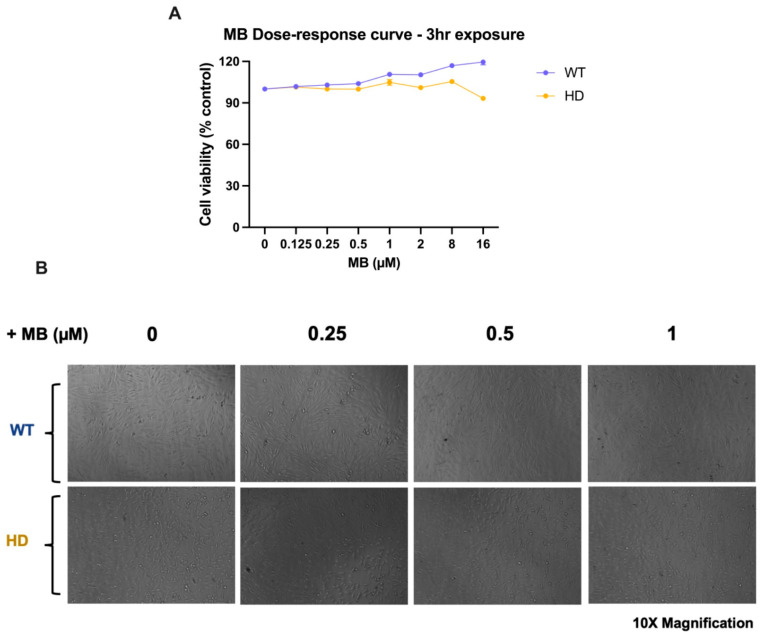

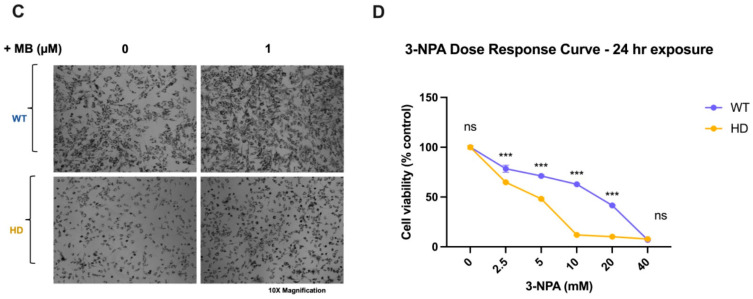
Striatal cell viability is susceptible to 3-NPA toxicity but remains unaffected by MB treatment. Cell viability was assessed with the MTT cell viability assay after treatment with MB (**A**) or 3-NPA (**D**). The average absorbance relative to the negative control for each genotype is plotted as a percentage of cell viability (±SEM). N = 4. (*** *p* < 0.001). (**B**) Representative phase contrast images of WT and HD cells treated with 0–1 µM MB for 3 h. (**C**) Representative formazan-stained brightfield images of WT and HD striatal cells following 3-h treatment with 0 or 1 µM MB. (**D**) 3-NPA dose–response curve following 24-h exposure to 0–40 mM 3-NPA in WT and HD cells. ns, not significant.

Changes in cell viability after MB pre-treatment for 3 h were established in WT and HD cells to determine if MB alone was sufficient to improve the survivability of HD cells to that of WT cells (basal differences) or if it could decrease neurotoxicity induced by the mutant HD gene. Cell viability was assessed following 3 h of pre-treatment in MB conditions. Based on the MB dose–response curves, we determined that treatment with 1 μM MB was the highest concentration that produced no significant effect on the viability of striatal cells (Figure 1A). MB pre-treatment was also sufficient to increase cell proliferation in both WT and HD cells without altering cell morphology (Figure 1B). Previous studies have reported increased susceptibility of mutant HD striatal cells against 3-NPA-induced neurotoxicity [43,44]. As a proof of concept, we generated a dose–response curve for 3-NPA, as an established pharmacological model of HD, after 24-h exposure (Figure 1C). As expected, HD striatal cells were more susceptible to the effects of 3-NPA than WT cells (Figure 1C) and experienced a more significant loss in cell viability. A 2-way ANOVA revealed significant effects of 3NPA, genotype, and the interaction between 3NPA and genotype treatment conditions (Figure 1D). The exacerbation of 3-NPA-induced cell loss in the mutant HD line supported our use of 3-NPA as a pharmacological model of HD. We chose 10 mM 3-NPA as the optimal concentration for subsequent experiments because it produced the most remarkable genotypic differences between WT and HD striatal cells after 24 h (Figure 1C).

### 2.2. Pre-Treatment with Mb Decreases Cell Death Induced by 3-Npa in HD and Wt Cells

**Figure 2:**
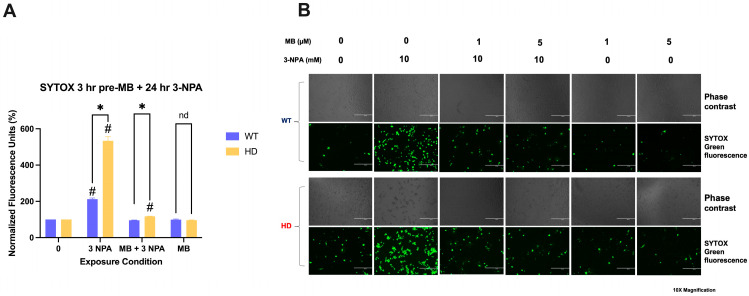
MB pre-treatment mitigates 3-NPA and HD-mediated neurotoxicity in striatal cells. (**A**) WT and HD were treated with 1 µM MB for 3 h, followed by exposure to 10 mM 3-NPA for 24 h. Cell death was assessed with the STYOX Green cell death assay. The average fluorescence relative to the negative control for each genotype is plotted as a percentage of cell death (±SEM, N = 4). (* *p* < 0.05 significant difference between genotypes at the indicated condition. # significant difference compared to the vehicle within genotype. Non-significance is indicated by ns. (**B**) Representative images at 10× magnification of WT and HD following 3-NPA exposure, as assessed by SYTOX Green cell death assay. Green positive cells indicate dead cells.

To investigate the potential therapeutic effect of MB against 3-NPA-induced neurotoxicity in striatal cells, MB pre-treatment reduced 3-NPA-induced cell death to baseline levels in HD cells, bringing the normalized fluorescence units from 533% following 3-NPA exposure to 117% when cells are treated with MB and then exposed to 3-NPA (Tukey’s adjusted *p* < 0.0001) (Figure 2A). WT cells also exhibited a complete rescue in cell death with MB pre-treatment, from 212% to 95% (Tukey’s adjusted *p* < 0.0001) (Figure 2A). Representative fluorescence images confirm MB-mediated therapeutic effects in both genotypes (Figure 2B).

### 2.3. MB Rescues Deficits in Mitochondrial Membrane Potential and Augments ATP Production

**Figure 3:**
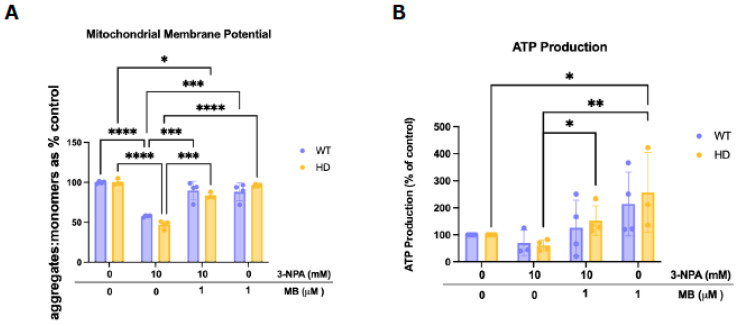
MB pre-treatment rescues HD and 3-NPA-mediated reduction in mitochondrial function in striatal cells. WT and HD cells were treated with 1 µM MB for 3 h, followed by exposure to 10 mM 3-NPA for 24 h. (**A**) Mitochondrial membrane health was assessed using the JC-1 assay. The ratio of JC-1 aggregates to monomers is graphed for each genotype as a percentage of the vehicle-treated control (±SEM). (**B**) Cellular ATP levels were similarly measured by a luminescence-based assay and are shown normalized to vehicle-treated control for each genotype (±SEM). N = 4. Statistical comparisons were performed by two-way ANOVA with Tukey’s multiple comparisons test. (* *p* < 0.05, ** *p* < 0.01, *** *p* < 0.001, **** *p* < 0.0001).

The JC-1 assay quantifies MMP by measuring the ratio of red fluorescence from dye aggregates (depolarization) to green fluorescence from monomers (hyperpolarization). Two-way ANOVA revealed a significant treatment effect (*F*(3,9) = 116.0, *p* < 0.0001). Exposure to 3-NPA significantly reduced the aggregate-to-monomer JC-1 ratio in both WT (mean ~57%) and HD (mean ~47%) cells compared to their respective vehicle controls (Tukey’s adjusted *p* < 0.0001), indicating a depolarized membrane and reduced electron transport chain activity. Treatment with MB alone preserved mitochondrial membrane potential close to control levels in both genotypes, and pre-treatment with MB and 3-NPA restored membrane potential to near baseline in WT (mean ~88%) and HD (mean ~96%) cells (Figure 3A). These results indicate that 3-NPA impairs mitochondrial membrane integrity, while MB treatment effectively preserves or restores mitochondrial membrane potential in WT and HD striatal cells. Cellular ATP levels (Figure 3B) measured after treatment with MB and/or 3-NPA showed a significant main effect of exposure condition (*F*(3,9) = 7.320, *p* = 0.0087) without a significant main effect of genotype or exposure × genotype interaction, as analyzed by a mixed-effects model. Following exposure to 3-NPA, ATP levels trended lower in both WT and HD cells compared to their vehicle-treated controls, though this did not reach significance. MB treatment alone modestly increased ATP levels compared to the vehicle. Pre-treatment with MB followed by 3-NPA exposure significantly increased ATP production compared to 3-NPA alone, particularly in HD cells (Tukey’s adjusted *p* = 0.0075), suggesting a protective or enhancing effect of MB on energy metabolism. These findings indicate that MB can help maintain or restore ATP levels following 3-NPA–induced energy impairment in both genotypes.

### 2.4. MB Alters 3-Npa-Induced Changes in Oxidative Stress-Related Proteins in Wt and HD Cells

**Figure 4:**
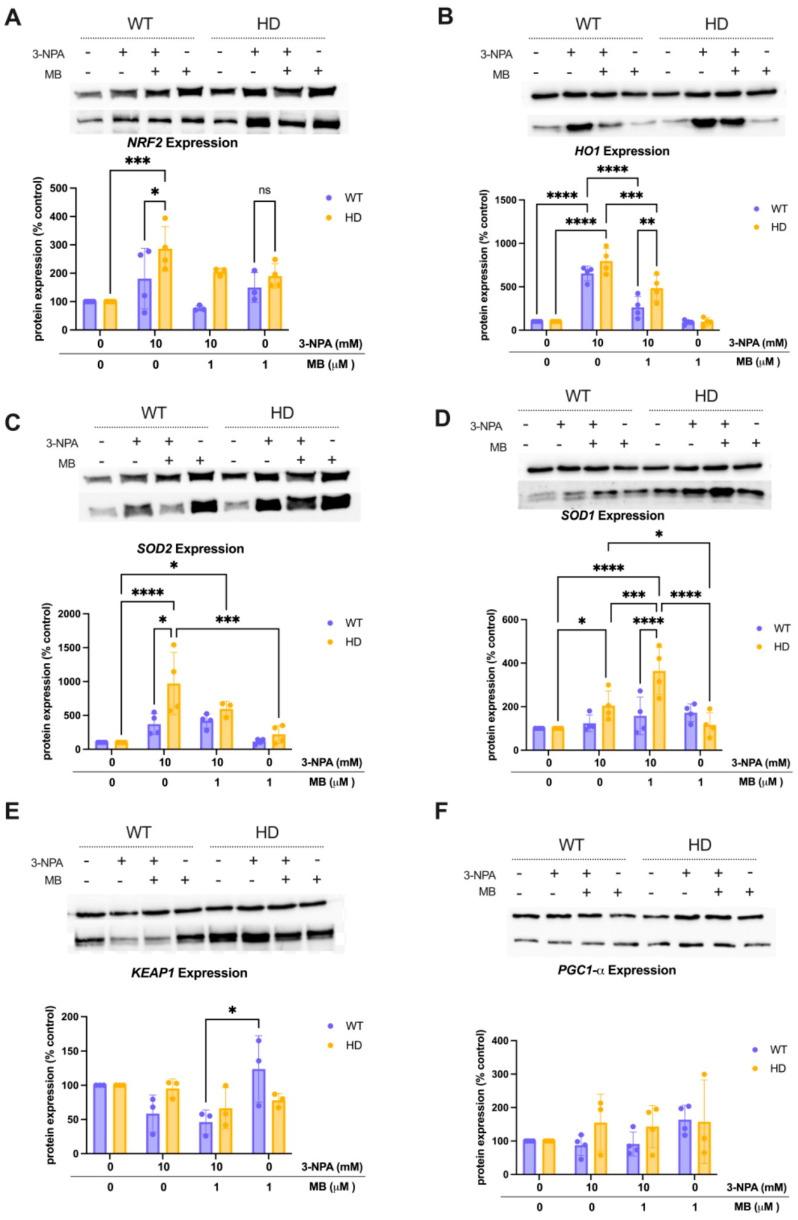
MB pre-treatment regulates 3-NPA-induced changes in oxidative stress proteins. WT and HD (N = 4) were treated with 1 μM MB for 3 h, followed by exposure to 10 mM 3-NPA for 24 h. Immunoblotting for (**A**) NRF2, (**B**) HO1, (**C**) SOD2, (**D**) SOD1, (**E**) KEAP1, and (**F**) PGC1-α proteins was normalized to respective beta-actin controls. The percentage (±SEM) relative to the negative control of each genotype is graphed. N = 4. Representative beta-actin and protein bands are shown in the images above. Tukey’s multiple comparison test compared the effect of exposure or treatment and genotypes (significant differences: * *p* < 0.05, ** *p* < 0.01, *** *p* < 0.001 and **** *p* < 0.0001). ns, not significant.

To examine the changes in oxidative stress response following exposure to 10 mM 3-NPA and pre-treatment with 1 μM MB, we measured the expression of key proteins involved in regulating oxidative stress. Several key enzymes of interest include the NRF2/KEAP1 pathway and their downstream effectors, such as HO-1 and SOD1/2. For NRF2 (Figure 4A), a mixed-effects model revealed a significant interaction between exposure condition and genotype (*p* = 0.0175), indicating differential NRF2 regulation depending on genotype and treatment. Specifically, 3-NPA significantly increased NRF2 expression in HD cells compared to WT controls (Tukey’s adjusted *p* = 0.0012), a pattern not significantly reversed by MB + 3-NPA, suggesting persistent stress signaling in HD despite MB. HO1 (Figure 4B), analyzed with two-way ANOVA, showed a strong main effect of exposure (*p* < 0.0001) and significant interaction (*p* = 0.0063), with robust increases in HO1 expression after 3-NPA in both genotypes (adjusted *p* < 0.0001), which were restored closer to baseline in HD cells only by MB + 3-NPA treatment, supporting MB’s role in regulating antioxidant responses. For SOD2 (Figure 4C), a mixed-effects model showed a significant interaction (*p* = 0.0357), with 3-NPA producing a marked elevation in SOD2 levels in HD cells (adjusted *p* < 0.0001), while MB + 3-NPA partially reduced this effect, though not significantly (*p* = 0.2358), indicating partial normalization of mitochondrial superoxide defense. Superoxide dismutase 1 (SOD1), a cytosolic antioxidant enzyme (Figure 4D), analyzed by two-way ANOVA, demonstrated a significant interaction (*p* < 0.0001), where 3-NPA elevated SOD1 in HD cells (*p* = 0.0119), while MB + 3-NPA treatment amplified SOD1 levels in HD cells (*p* = 0.0006), suggesting a possible compartment-specific response that may be more effectively regulated in the mitochondria. KEAP1 (Figure 4E), tested with two-way RM ANOVA, showed a significant main effect of exposure (*p* = 0.0177) but no genotype interaction.

In contrast, PGC1-α (Figure 4F), a master regulator of mitochondrial biogenesis, revealed no significant main effects or interactions (all *p* > 0.18) and no pairwise differences by Tukey’s test, suggesting stable PGC1-α expression across treatment groups. Together, these results demonstrate that 3-NPA broadly induces oxidative and proteostatic stress responses in HD striatal cells, with evidence of genotype-specific vulnerability in antioxidant pathways. Importantly, MB pre-treatment was able to reduce these maladaptive responses across most proteins tested, highlighting its potential as a modulator of oxidative and mitochondrial stress in HD cellular models. In contrast, PGC1-α (Figure 4F), a master regulator of mitochondrial biogenesis, revealed no significant main effects or interactions (all *p* > 0.18) and no pairwise differences by Tukey’s test, suggesting stable PGC1-α expression across treatment groups.

### 2.5. MB Alters the 3-Npa-Induced Changes in the Expression of Proteins Involved in Regulating Mitochondrial Dynamics

**Figure 5:**
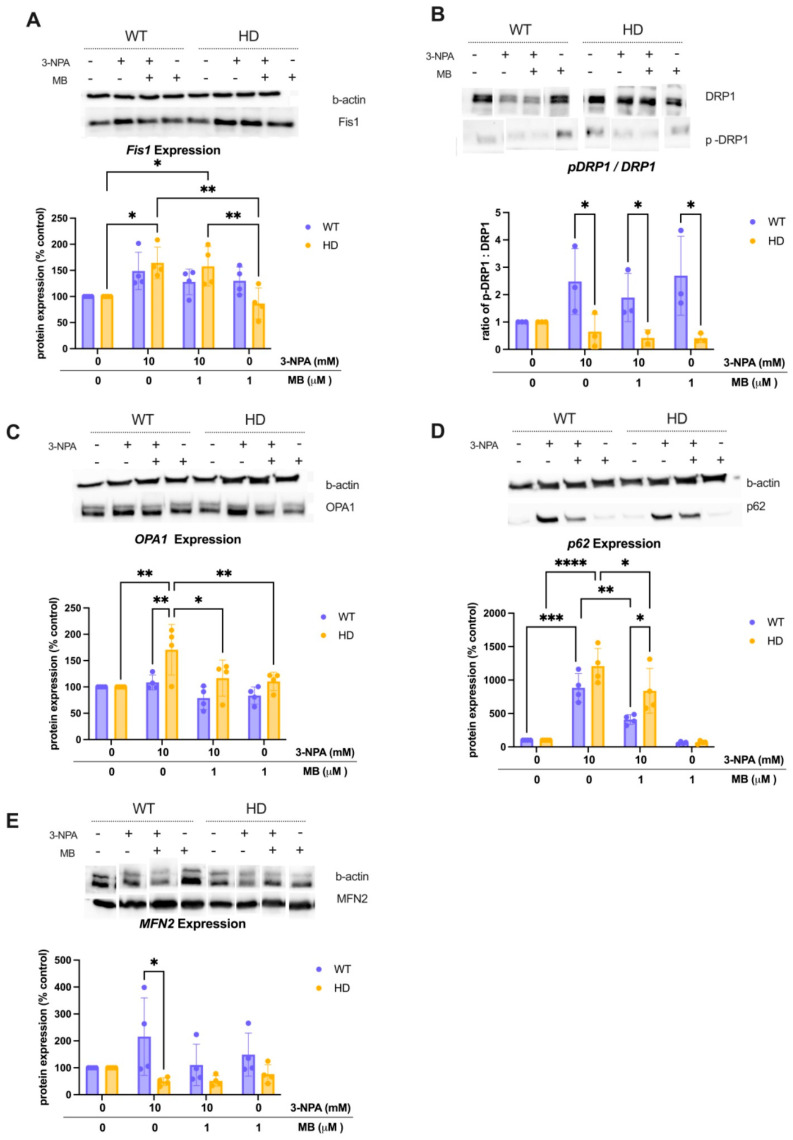
MB pre-treatment modulates 3-NPA-induced changes in mitochondrial dynamics proteins. WT and HD striatal cells were treated with 1 μM MB for 3 h, followed by exposure to 10 mM 3-NPA for 24 h. Immunoblotting for (**A**) Fis1, (**B**) Ratio of p-DRP1/DRP1, (**C**) OPA1, (**D**) p62, and (**E**) MFN2 proteins was normalized to respective beta-actin controls. The percentage (±SEM) relative to the negative control of each genotype is graphed. N = 4. Representative beta-actin and protein bands are shown in the images above. Tukey’s multiple comparison test compared the effect of exposure/treatment and genotypes (significant differences: * *p* < 0.05, ** *p* < 0.01, *** *p* < 0.001, and **** *p* < 0.0001).

To further elucidate the effect of exposure to 10 mM 3-NPA and pre-treatment with 1 μM MB on mitochondrial function, we examined mitochondrial remodeling and biogenesis markers in WT and HD striatal cells. Expression of Fission1 (Fis1) (Figure 5A), assessed by two-way repeated-measures ANOVA, demonstrates a significant main effect of exposure condition (*p* = 0.0055, *F*(3,9) = 8.45) and a significant exposure × genotype interaction (*p* = 0.0154), but no main effect of genotype. Post hoc Tukey tests revealed that 3-NPA significantly increased Fis1 expression in HD cells relative to the WT baseline (*adjusted p* = 0.0107), and that MB pre-treatment + 3-NPA does not significantly alter the effect of 3-NPA alone. To examine a mediator of mitochondrial fission, we quantified Dynamin-related protein (DRP1) activation as the ratio of phosphorylated DRP1 to total DRP1 expression in WT and HD cells (Figure 5B). There was no significant effect on DRP1 activation in any of the exposure groups. However, genotype exerted a significant main effect (*F*(1,18) = 14.27, *p* = 0.0014), with WT cells consistently exhibiting higher DRP1 activation compared to HD cells across all treatments. These findings suggest that DRP1 activation is primarily genotype-dependent, and 3-NPA treatment alone does not significantly alter DRP1 activation levels.

OPA1 (Figure 5C), a key regulator of mitochondrial inner membrane fusion, was analyzed by two-way ANOVA and showed a significant main effect of exposure (*F*(3,9) = 11.31, *p* = 0.0021) and a significant interaction between genotype and exposure (*p* = 0.0245). 3-NPA treatment significantly increased OPA1 levels in HD cells (*p* = 0.0024), and MB + 3-NPA pre-treatment restored these elevations toward baseline (post hoc *p* values < 0.005). Additionally, expression levels of Mitofusin-2 (MFN2), a GTPase that mediates mitochondrial fusion at the outer membrane, were measured (Figure 5E). Two-way repeated measures ANOVA showed no significant main effect of exposure (*F*(3,9) = 1.84, *p* = 0.21) or genotype (*F*(1,3) = 3.76, *p* = 0.15) on MFN2 levels. However, post hoc multiple comparisons revealed that MFN2 expression was significantly higher in WT cells treated with 3-NPA compared to HD cells treated with 3-NPA, and this difference was not significantly altered by MB pre-treatment.

Lastly, p62 (Figure 5D) showed a significant interaction with two-way ANOVA (*p* = 0.0186), with 3-NPA dramatically increasing p62 accumulation in HD cells (adjusted *p* < 0.0001), consistent with impaired autophagic flux under mitochondrial toxin challenge. However, MB + 3-NPA restored p62 levels to those similar to vehicle-treated cells in both genotypes (*p* > 0.9999). Together, these results indicate that 3-NPA disrupts the mitochondrial fission–fusion balance in HD cells by increasing Fis1, DRP1, and OPA1 levels, while MB pre-treatment mitigates these alterations in OPA1 and p62 expression, supporting a potential role of MB in normalizing mitochondrial dynamics during mitochondrial stress induced by 3-NPA.

### 2.6. Mutant HD Regulates Basal Protein Levels of Oxidative Stress and Mitochondrial Dynamics Signaling Proteins

**Figure 6:**
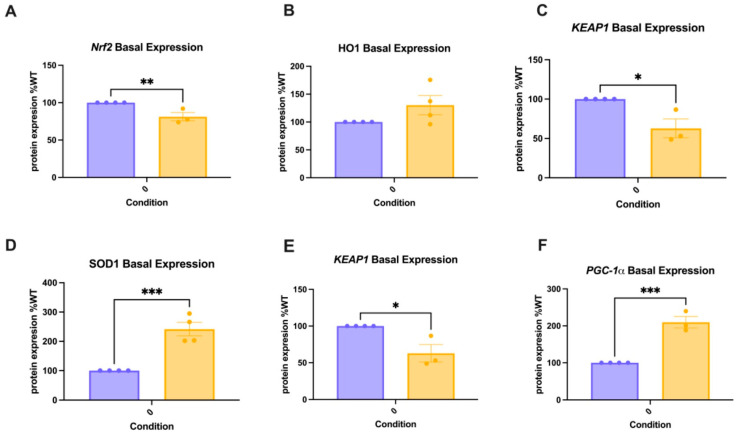

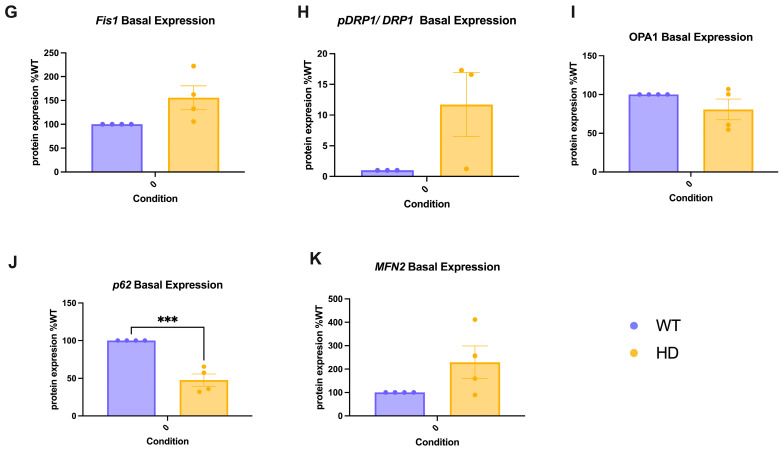
Basal differences in the expression of oxidative stress and mitochondrial biogenesis proteins. WT and HD were treated with 1 µM MB for 3 h, followed by exposure to 10 mM 3-NPA for 24 h. Immunoblotting for basal (**A**) NRF2, (**B**) HO1, (**C**) SOD2, (**D**) SOD1, (**E**) KEAP1, (**F**) PGC1-α, (**G**) Fis1, (**H**) p-DRP1: DRP1, (**I**) OPA1, (**J**) p62, and (**K**) MFN2 proteins were normalized to their respective beta-actin controls. N = 4. Percentage (±SEM) relative to the negative control of the WT genotype is graphed. Student’s *t*-test compared the effect of Exposure and Genotypes (significant differences: * *p* < 0.05, ** *p* < 0.01, and *** *p* < 0.001).

To determine the genotypic differences in the expression of oxidative stress and mitochondrial biogenesis proteins in WT compared to HD cells, the basal levels of oxidative stress and mitochondrial proteins were measured. The protein expression levels in the untreated HD cells were normalized to those in the WT cells. A Student’s *t*-test was conducted for each protein to compare expression in WT vs. HD cells. The basal levels of NRF2 were lower in HD cells as compared to WT cells (difference between means −18.76 ± 4.636, *p* = 0.0099). SOD2 levels were also lower in HD cells as compared to WT (difference between means −37.15 ± 10.02, *p* = 0.0139), indicating a potential weakening of the primary mitochondrial defense systems against oxidative stress in HD cells. Conversely, HD cells exhibited significantly elevated levels of oxidative stress proteins SOD1 (Figure 6D) (241.6 ± 23.2 vs. 100.0 ± 0; *p* = 0.0009) and PGC1-α (Figure 6F) (209.8 ± 13.0 vs. 100.0 ± 0; *p* = 0.0004), suggesting a compensatory antioxidant response.

Total mitochondrial fission protein DRP1 was reduced in HD cells, but the pDRP1/DRP1 ratio (a measure of activated DRP1) was markedly higher in HD cells (16.4 ± 4.9) compared to WT cells (Figure 6H), though this was not statistically significant. KEAP1 (Figure 6E), an inhibitor of NRF2-mediated signaling, was elevated in HD cells (199.1 ± 36.9 vs. 100.0 ± 0; *p* = 0.0436). Expression levels of P62 (Figure 6J), a ubiquitin-binding protein that mediates mitophagy, were significantly reduced in HD cells (47.7 ± 8.2 vs. 100.0 ± 0; *p* = 0.0007), suggesting impaired baseline mitophagy capacity. At baseline, MFN2 expression was higher in HD cells (229.5 ± 69.3) compared to WT cells, indicating a trend towards elevated outer membrane fusion protein levels due to mHTT (Figure 6K).

## 3. Discussion

The pathology of HD has yet to be fully clarified, but defects in energy metabolism have been reported substantially in both animal models of HD and HD patients [13,29]. Neurons have high energy demands, so alterations to pathways that supply energy leave these cells especially vulnerable [29]. Mutant HTT potentiates ROS production in striatal cells through impairments to mitochondrial function and antioxidant buffering capacity, as indicated by studies that report increased ROS in striatal cells expressing mHTT and oxidative stress [10,15,45,46,47]. The mitochondrial membrane potential in HD cells is depolarized, limiting energy production [22,48]. Disruption of complex II limits energy production and increases ROS, which may further the pathogenesis of HD [49].

This study confirms findings that 3-NPA, a pharmacological inducer of HD, exacerbates the effects of the mutant HTT and causes a more significant decrease in cell viability in an established striatal cell model of HD compared to healthy cells, a finding supported by 3-NPA’s extensive use as a pharmacological model of HD in vivo and in vitro models [28]. 3-NPA offers an advantage in studying how the mutated HTT and an aggravated chemical event cooperatively mediate impaired mitochondrial function. Additionally, we report a novel neuroprotective role for MB, the current treatment for methemoglobinemia and sepsis, in HD.

10 mM 3-NPA elicited the most pronounced genotypic difference in cell viability between HD and WT cells, effectively demonstrating the heightened susceptibility of HD cells to inhibition of mitochondrial complex II. Thus, 10 mM 3-NPA was used in subsequent experiments to examine how a genetic inducer of HD (mHTT expression), coupled with an environmental inducer (the neurotoxin 3-NPA), synergistically disrupts processes in striatal cells. This model enabled us to simulate an aggravated pathological environment to mimic scenarios where genetic predisposition (expressing mHTT) interacts with environmental insults (exposure to neurotoxin) to accelerate neurodegeneration. We hypothesized that MB would attenuate the effects of this neurotoxin to a greater extent in HD cells as compared to healthy, WT cells. We determined that 1 μM was the optimal concentration to cause no significant genotypic difference in cell viability between WT and HD cells (Figure 1A,B), indicating that MB improved overall metabolic capability similarly in both genotypes at this concentration. MB ameliorated cell death in both genotypes (Figure 3), though this rescue was more notable in HD cells, which were significantly impacted by 3-NPA, unlike the WT cells. To explore mechanisms that precede changes in cell viability, mitochondrial function was assessed. Gomez-Sequeda and colleagues (2024) have demonstrated that MB increases cell viability and mitochondrial activity in a cellular model of familial AD [37], and Liu et al. (2024) have shown significant ROS inhibition by MB in a tauopathy-like cellular model [38]. In the case of mitochondria complex I inhibition, MB has been hypothesized to rescue mitochondrial membrane potential by acting as an alternative electron carrier, bypassing the inhibited complex and facilitating electron transport [50]. Wen and colleagues (2011) demonstrate that MB transfers electrons from complex I to cytochrome c, even when these complexes are inhibited [51]. We report that MB eliminates the genotypic difference in membrane potential resulting from 3-NPA exposure (Figure 3A), demonstrating that it recovers mitochondrial activity that has been reduced by mutant huntingtin (mHTT) and 3-NPA. Given that the reported mitochondrial membrane potential is the primary indicator of oxidative phosphorylation [52], we demonstrate that MB attenuates the effect of 3-NPA on overall mitochondrial function in a striatal cell model of HD. Similarly, while 3-NPA reduced ATP production, MB showed a trend towards rescuing ATP levels, suggesting partial restoration of oxidative phosphorylation capacity (Figure 3B).

Given the role of mitochondrial dysfunction in oxidative stress, we examined the role of oxidative stress pathways in MB-mediated neuroprotection. NRF2 is a transcription factor that regulates the expression of antioxidant and detoxification enzymes, and KEAP1 is a negative regulator of NRF2 [53]. When cells undergo oxidative stress, NRF2 is stabilized and translocated to the nucleus, where it binds to the antioxidant response element (ARE) and induces the expression of genes encoding antioxidant enzymes and detoxification proteins [53]. SOD1 is an antioxidant enzyme that catalyzes the dismutation of superoxide radicals, while HO1 is involved in detoxification processes as a downstream target of NRF2 activation [54]. mHTT exacerbates oxidative stress and causes mitochondrial dysfunction, increasing the demand for these proteins to counteract oxidative damage [7]. mHTT can impair the NRF2-KEAP1 pathway, further compromising the cells’ ability to respond to oxidative stress [8]. In a PC12 model of HD, which expresses an exon 1 fragment of huntingtin and represents an early stage of cellular pathology, there was an increase in NRF2-responsive genes [55], warranting a closer examination of the NRF2 pathway in HD.

Interestingly, in striatal cells expressing mHtt or wild-type Htt, Jin et al. (2013) report that disrupted NRF2 signaling is not due to its expression levels [9], suggesting that an increase in NRF2 levels would not necessarily indicate an improved response to oxidative stress. We do report genotype-specific vulnerabilities through differences in basal protein expression. HD cells exhibited lower basal levels of NRF2 and mitochondrial SOD2, indicating that their antioxidant defense system is intrinsically compromised. Based on previous studies examining the effect of MB on the NRF2 pathway, we anticipated that MB would upregulate this pathway in cells exposed to a complex inhibitor. Bhurtel et al. demonstrated a clear activation of NRF2 by MB in dopaminergic cells exposed to MPTP, a complex I inhibitor [50]. However, MB pre-treatment did not significantly alter NRF2 levels (Figure 4A). This finding may be consistent with the disrupted NRF2 activity described in HD models, which could reflect dysregulation of NRF2 translocation or function, rather than expression alone. KEAP1, the regulator of NRF2, is not significantly altered by exposure to 3-NPA alone, or MB pre-treatment followed by 3-NPA exposure (Figure 4E), which provides limited information given its function. When it releases NRF2, KEAP1 is not degraded, so we cannot assume that a change in its expression indicates a change in NRF2. Thus, we examined the expression of antioxidant enzymes downstream of NRF2 signaling to determine how mHTT might impair NRF2 signaling and function.

Cells expressing mHTT are under increased stress due to the combination of the mutation and exposure to 3-NPA, which will over-activate various signaling pathways, including those involved in oxidative stress responses. We report a significant increase in HO1 expression in both WT and HD cells following 3-NPA exposure. MB attenuates the increase in HO1 expression in both genotypes (Figure 4B). We can deduce that 3-NPA elicits a need for antioxidant enzymes, such as HO-1, and that the reduction observed with MB pre-treatment indicates the reduced need for HO-1. One mechanism by which MB exerts its antioxidant effects is through its ability to act as a redox cycler, accepting electrons from reducing equivalents and thereby preventing the formation of reactive oxygen species (ROS) [30]. Additionally, MB has been shown to enhance mitochondrial function and reduce oxidative damage to mitochondria [33,56], which can help maintain cellular energy production and reduce the overall oxidative burden on the cell, accounting for the decrease in HO1 observed when cells are pre-treated with MB compared to those exposed only to 3-NPA. Contrary to previously published literature, which shows that an increase in HO-1 levels in dopaminergic cells exposed to a complex I inhibitor results in diminished MB protection [50], our results indicate that MB reduces 3-NPA-induced expression of HO-1.

Additionally, MB modulated SOD1 and SOD2 expression, further suggesting that MB supports redox balance not solely by promoting NRF2 signaling but by directly limiting ROS formation at the mitochondria and indirectly shaping downstream enzyme responses. SOD1, a cytosolic antioxidant enzyme, is further upregulated by pre-treatment with MB (Figure 4D). Changes in cytosolic SOD1 were not significant in WT cells, suggesting that the upregulation of SOD1 is possibly specific to the mutant HTT, rather than being a result of complex II inhibition by 3-NPA. Conversely, SOD2 levels reflect those of HO1 and are reduced to baseline by pre-treatment with MB (Figure 4C). In a Tau P301S mouse model of AD, a diet supplemented with MB resulted in significantly increased SOD2 levels, which reduced oxidative stress [42]. However, our data demonstrate a normalization of oxidative defenses in the mitochondria with MB pre-treatment. The divergence in SOD2 and SOD1 may indicate compartment-specific regulation, with MB exerting a greater effect in the mitochondria. A similar duality, describing MB as both an antioxidant and a mild prooxidant, is described by Gureev et al. (2019) [41]. We report basal expression level differences in PGC-1α (Figure 6F), with HD cells expressing significantly higher levels than WT. This aligns with the increased basal level expression of antioxidant response proteins KEAP1 and SOD1 (Figure 6D,E) in HD cells, given the role of PGC-1α in activating these pathways [34]. These elevations may suggest an intrinsic compensatory response to persistent cellular stress in HD cells. Interestingly, we did not observe any significant changes in PGC-1α expression in either genotype across any treatment group (Figure 6G). However, the trends indicate an increased expression of PGC-1α in HD cells across all conditions. Weydt and colleagues reported that 3-NPA reduced mitochondrial membrane potential in ST*Hdh* cells, but PGC-1α-transfected cells maintained mitochondrial membrane potential, indicating that PGC-1-α bolstered mitochondrial function [57]. Further, Cui et al. (2006) showed that the loss of PGC-1α increased the susceptibility of their HD mouse model to 3-NPA toxicity [16]. In our study design, temporal differences in the changes to PGC-1α transcription in response to 3-NPA may be preventing us from observing a significant effect of either 3-NPA exposure or pre-treatment with MB.

Overall, the dysregulation of these oxidative stress-related proteins by 3-NPA and mHTT highlights the complex interplay between mitochondrial dysfunction, oxidative stress, and neurodegeneration in HD. MB appears to reduce the need for certain antioxidant enzymes by enhancing mitochondrial function.

Oxidative stress and mitochondrial dynamics are intricately linked, with oxidative stress influencing mitochondrial function, and dysfunctional mitochondrial dynamics contributing to the development of oxidative stress [58,59]. Thus, it is unsurprising that disruptions to oxidative phosphorylation would be linked to changes to the proteins that regulate these mitochondrial dynamics processes in HD. In vitro and in vivo models of HD have demonstrated an increase in crucial fission proteins, including DRP1 and Fis1 [22,24,60]. Guo et al. (2013) report an increase in DRP1 and mitochondrial fragmentation in pluripotent stem cells derived from HD patients, suggesting a role for fission in HD pathology [22]. The basal ratio of pDRP1 to total DRP1 was elevated in cells expressing mutant HTT (Figure 6H), indicating increased DRP1 phosphorylation at rest. However, we did not observe a further increase in DRP1 activation in response to 3-NPA in either genotype (Figure 5B). In contrast, Fis1 expression significantly increased in HD cells following 3-NPA treatment, whereas WT cells showed a non-significant increase (Figure 5A). These data suggest that genotypic differences driven by mutant HTT influence DRP1 activation, and thus the initiation of fission, while 3-NPA specifically affects Fis1 expression, which is one of the proteins that recruits DRP1 to the mitochondrial membrane. Moreover, pre-treatment with MB did not rescue these alterations in mitochondrial fission protein expression (Figure 5A,B).

Optic atrophy 1 protein (OPA1) is a regulator of mitochondrial fusion. One study reported that OPA1 could restore lymphoblast fragmentation from HD patients and striatal cell lines [24]. Interestingly, we report an increase in OPA1 in our mutant HD striatal cells following exposure to 3-NPA and attenuation of this increase by MB pre-treatment (Figure 5C). MFN2, which mediates outer membrane fusion, displayed elevated basal expression in HD cells and significant genotype differences under 3-NPA, but no significant main effect of exposure (Figure 5E). This pattern suggests that MFN2 dysregulation is present at baseline in HD cells, whereas OPA1 responds dynamically to metabolic stress. Basal MFN2 elevation, alongside inducible OPA1 regulation with 3-NPA, suggests that outer and inner membrane fusion components are differentially dysregulated in HD, pointing to a layered control of mitochondrial dynamics that warrants further exploration. While OPA1 expression has not been reported in the context of MB in in vitro models, we can infer that improving the bioenergetics of the mitochondria through MB will have downstream effects on protecting the greater function of the organelle, including reducing the dysregulation of these proteins. It is to be expected that changes to oxidative stress would trigger mitochondria to undergo fusion and fission to maintain proper function. Given the mechanism of 3-NPA, we anticipated changes in the expression of these proteins. The HD cells, which exhibit more striking changes in most cases, indicate further dysregulation in the presence of the mutated HTT. While we report an effect of 3-NPA on key fusion and fission proteins DRP-1, OPA1, and Fis1 (Figure 5A–C), our data indicate that MB protects mitochondrial function, but its impact on fission-fusion regulation is less robust.

To further assess the mechanisms by which MB might regulate mitochondrial function, we measured the expression of proteins associated with the clearance of unhealthy mitochondria. The ubiquitin-binding protein p62 plays a role in oxidative stress response by mediating autophagy [61]. In mitophagy, p62 is a mediator that interacts with LC3 on the autophagosome, targeting damaged mitochondria for degradation [61]. Basal expression of p62 was markedly lower in HD cells (Figure 6J), indicating possible impairment of autophagic clearance of damaged mitochondria. As expected, 3-NPA-induced stress in both genotypes increases p62 expression, which is partially recovered by pre-treatment with MB in both genotypes (Figure 5D), reflecting another potential mechanism by which MB preserves mitochondrial homeostasis, not only by directly improving mitochondrial function.

**Figure 7:**
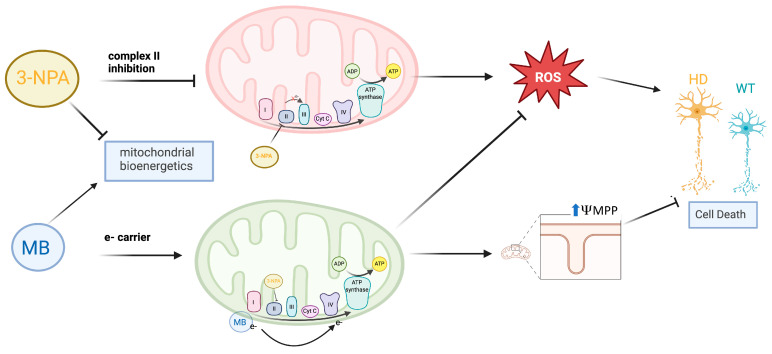
A proposed model for MB mediated neuroprotection against 3-NPA-induced neurotoxicity and neurodegeneration in striatal cells. Specifically, MB restores mitochondrial bioenergetics and attenuates oxidative stress pathways to augment striatal cell health and viability.

Altogether, these results show that MB preserves mitochondrial function, both structurally and energetically, by restoring membrane potential and ATP synthesis, and by protecting against cell death in a chemical-genetic HD model. Its benefits appear to extend to downstream pathways that mediate oxidative stress, reducing excessive antioxidant responses such as HO1 while enhancing SOD enzymes. MB also partially modulates mitochondrial dynamics, reversing the 3–NPA–induced increases in OPA1; however, its effects on fission proteins were more limited. We observe specific effects more strongly in HD cells than in WT cells with MB pre-treatment, indicating that the protective effects of MB may be mediated by the HD gene. In conclusion, MB shows promise in mediating toxicity induced by 3-NPA, a pharmacological inducer of HD in our wild-type and mutant HTT-expressing striatal cells. By restoring mitochondrial membrane potential and restoring some of the antioxidant defenses and mitochondrial biogenesis alterations triggered by 3-NPA, MB demonstrates mechanisms that counteract mitochondrial dysfunction (Figure 7). Despite the novelty of these findings, all experiments were performed in vitro using an established immortalized cell line from a knock-in transgenic HD mouse model, rather than in primary neurons or animal models, which may exhibit different survival and antioxidant activities. Although it is beyond the scope of this paper, future studies will examine the therapeutic effects of MB in animal models of HD to further translate these novel findings. Given the well-established role of oxidative stress and mitochondrial impairments in HD, and the novel neuroprotective findings discussed in this paper, future studies should consider exploring methylene blue as a potential disease-altering therapy.

## 4. Materials and Methods

### 4.1. Cell Model

We used an established striatal cell line from a knock-in transgenic mouse model that expresses either wild-type HTT (WT) with 7 polyglutamine repeats (STHdhQ7/Q7) or homozygous mutant HTT (HD) with 111 polyglutamine repeats (STHdhQ111/Q111) [9,44,62]. Immortalized striatal WT and HD cell lines were obtained from Coriell Institute for Medical Research (Camden, NJ, USA). Cells were cultured in DMEM with 4.5 g/L glucose supplemented with 10% FBS, 1% P/S, 400 μg/mL G418, and 1% GlutaMAX at 33 °C with 5% CO_2_.

### 4.2. Drug Treatment and Morphological Observation

WT and HD were seeded overnight and treated with MB [0–20 µM] for 3 h, or 3-NPA [0–40 mM] for 24 h, and the effective dosages were determined based on dose–response curves generated from MTT metabolic activity assay. Cell morphology was photographed and analyzed using an EVOS brightfield microscope (Invitrogen, Thermo Fisher, Waltham, MA, USA). For subsequent investigations, equal numbers of WT and HD cells were seeded overnight and treated with one of the following conditions: (i) 1 µM for 3 h; (ii) 10 mM 3-NPA for 24 h; or (iii) pre-treated with MB followed by 3-NPA exposure.

### 4.3. Cell Death Assay

Equal numbers of ST*Hdh*Q111/Q111 and ST*Hdh*Q7/Q7 were seeded overnight and then treated with (i) 1 μM MB for 3 h, (ii) 10 mM 3-NPA for 24 h, or (iii) MB and 3-NPA sequentially. Cell death was measured using the SYTOX Green assay (SYTOX Green from Life Technologies, Grand Island, NY, USA). Briefly, SYTOX green dye penetrates only compromised cells and fluoresces when bound to nucleic acids via intercalation. Fluorescence was measured with a Synergy HT multi-mode microplate reader (BioTek Instruments, Inc, Winooski, VT, USA) at 485 nm excitation and 535 emission wavelengths. Changes in fluorescence are proportional to cell death. Representative images were captured with an EVOS fluorescence microscope (Invitrogen).

### 4.4. Mitochondrial Membrane Potential

Mitochondrial membrane potential (MMP) is crucial for ATP production and serves as an indicator of proper mitochondrial bioenergetics. MMP was determined using the JC-1 assay, which measured the shift in fluorescence from green (depolarization) to red (hyperpolarization). Fluorescent signals were measured and quantified using a Synergy HT microplate reader (BioTek Instruments Winnooski, Winooski, VT, USA).

### 4.5. ATP Assay

Change in cellular ATP production was assessed using an ATP detection assay (Cayman Chemical, Ann Arbor, MI, USA). Briefly, luciferase uses ATP to convert luciferin to oxyluciferin and emit light. The luminescence intensity is directly proportional to the amount of cellular ATP. Following cell treatments, changes in luminescence were measured using a Synergy HT microplate reader.

### 4.6. Immunoblotting

Whole-cell lysates were prepared from cells following drug treatments using RIPA buffer, 1X protease, and phosphatase inhibitor cocktails. Samples were standardized using the DC protein assay, and equal amounts of proteins from each sample were loaded into a 10% SDS-polyacrylamide gel. Proteins were separated by SDS-PAGE and transferred to a nitrocellulose membrane using either wet or semi-dry transfer methods. Non-specific binding sites were blocked with 3% BSA for 1 h at room temperature, and the membranes were incubated overnight at 4 °C with primary antibodies directed against HO1, SOD1, SOD2, KEAP1, NRF2, p62, Fis1, DRP1, OPA1, PGC1-ɑ, and β-Actin (Cell Signaling Technology, Danvers, MA, USA). Blots were incubated with appropriate HRP-conjugated secondary antibodies, and bands were visualized using luminol-based chemiluminescent substrates (Thermo Scientific, Waltham, MA, USA) and imaged using a ChemiDoc Touch imaging system (BioRad, Hercules, CA, USA). The integrated density of protein bands was measured using ImageStudioLite software, version 5.2.5 (LI-COR, Lincoln, NE, USA).

### 4.7. Statistical Analysis

Data were graphed and analyzed using GraphPad PRISM 10.4.1 software. Values are expressed as the mean ± SEM and analyzed using one-way and three-way ANOVA with Tukey’s post hoc test to determine differences and interactions between genotype, MB pre-treatment, and 3-NPA exposure. Mixed-effect analysis was used instead of three-way ANOVA when values were omitted. Values of *p* < 0.05 are considered statistically significant.

## Data Availability

The authors confirm that the data supporting the findings of this study are available within the article and its Supplementary Materials.

## Supplementary Materials

The following supporting information can be downloaded at: https://www.mdpi.com/article/10.3390/ijms262110672/s1.

## References

[1] Ghosh R., Tabrizi S.J. (2018). Clinical Features of Huntington’s Disease. Adv. Exp. Med. Biol..

[2] Nopoulos P.C. (2016). Huntington Disease: A Single-Gene Degenerative Disorder of the Striatum. Dialogues Clin. Neurosci..

[3] Sharp A.H., Loev S.J., Schilling G., Li S.H., Li X.J., Bao J., Wagster M.V., Kotzuk J.A., Steiner J.P., Lo A. (1995). Widespread Expression of Huntington’s Disease Gene (IT15) Protein Product. Neuron.

[4] Ratovitski T., Chighladze E., Arbez N., Boronina T., Herbrich S., Cole R.N., Ross C.A. (2012). Huntingtin Protein Interactions Altered by Polyglutamine Expansion as Determined by Quantitative Proteomic Analysis. Cell Cycle.

[5] Schulte J., Littleton J.T. (2011). The Biological Function of the Huntingtin Protein and Its Relevance to Huntington’s Disease Pathology. Curr. Trends Neurol..

[6] Dayalu P., Albin R.L. (2015). Huntington Disease: Pathogenesis and Treatment. Neurol. Clin..

[7] Singh A., Kukreti R., Saso L., Kukreti S. (2019). Oxidative Stress: A Key Modulator in Neurodegenerative Diseases. Molecules.

[8] Sawant N., Morton H., Kshirsagar S., Reddy A.P., Reddy P.H. (2021). Mitochondrial Abnormalities and Synaptic Damage in Huntington’s Disease: A Focus on Defective Mitophagy and Mitochondria Targeted Therapeutics. Mol. Neurobiol..

[9] Jin Y.N., Yu Y.V., Gundemir S., Jo C., Cui M., Tieu K., Johnson G.V.W. (2013). Impaired Mitochondrial Dynamics and Nrf2 Signaling Contribute to Compromised Responses to Oxidative Stress in Striatal Cells Expressing Full-Length Mutant Huntingtin. PLoS ONE.

[10] Ribeiro M., Rosenstock T.R., Oliveira A.M., Oliveira C.R., Rego A.C. (2014). Insulin and IGF-1 Improve Mitochondrial Function in a PI-3K/Akt-Dependent Manner and Reduce Mitochondrial Generation of Reactive Oxygen Species in Huntington’s Disease Knock-in Striatal Cells. Free Radic. Biol. Med..

[11] Damiano M., Galvan L., Déglon N., Brouillet E. (2010). Mitochondria in Huntington’s Disease. Biochim. Biophys. Acta.

[12] Tucker D., Lu Y., Zhang Q. (2018). From Mitochondrial Function to Neuroprotection-an Emerging Role for Methylene Blue. Mol. Neurobiol..

[13] Browne S.E., Bowling A.C., MacGarvey U., Baik M.J., Berger S.C., Muqit M.M., Bird E.D., Beal M.F. (1997). Oxidative Damage and Metabolic Dysfunction in Huntington’s Disease: Selective Vulnerability of the Basal Ganglia. Ann. Neurol..

[14] Damiano M., Diguet E., Malgorn C., D’Aurelio M., Galvan L., Petit F., Benhaim L., Guillermier M., Houitte D., Dufour N. (2013). A Role of Mitochondrial Complex II Defects in Genetic Models of Huntington’s Disease Expressing N-Terminal Fragments of Mutant Huntingtin. Hum. Mol. Genet..

[15] Johri A., Chandra A., Flint Beal M. (2013). PGC-1\alpha, Mitochondrial Dysfunction, and Huntington’s Disease. Free Radic. Biol. Med..

[16] Cui L., Jeong H., Borovecki F., Parkhurst C.N., Tanese N., Krainc D. (2006). Transcriptional Repression of PGC-1alpha by Mutant Huntingtin Leads to Mitochondrial Dysfunction and Neurodegeneration. Cell.

[17] Johri A., Starkov A.A., Chandra A., Hennessey T., Sharma A., Orobello S., Squitieri F., Yang L., Beal M.F. (2011). Truncated Peroxisome Proliferator-Activated Receptor-\gamma Coactivator 1\alpha Splice Variant Is Severely Altered in Huntington’s Disease. Neurodegener. Dis..

[18] Kim J., Moody J.P., Edgerly C.K., Bordiuk O.L., Cormier K., Smith K., Beal M.F., Ferrante R.J. (2010). Mitochondrial Loss, Dysfunction and Altered Dynamics in Huntington’s Disease. Hum. Mol. Genet..

[19] Pernas L., Scorrano L. (2016). Mito-Morphosis: Mitochondrial Fusion, Fission, and Cristae Remodeling as Key Mediators of Cellular Function. Annu. Rev. Physiol..

[20] Manczak M., Reddy P.H. (2015). Mitochondrial Division Inhibitor 1 Protects against Mutant HuntingtinInduced Abnormal Mitochondrial Dynamics and Neuronal Damage in Huntington’s Disease. Hum. Mol. Genet..

[21] Yin X., Manczak M., Reddy P.H. (2016). Mitochondria-Targeted Molecules MitoQ and \SS31 Reduce Mutant Huntingtin-Induced Mitochondrial Toxicity and Synaptic Damage in Huntington’s Disease. Hum. Mol. Genet..

[22] Guo X., Disatnik M.-H., Monbureau M., Shamloo M., Mochly-Rosen D., Qi X. (2013). Inhibition of Mitochondrial Fragmentation Diminishes Huntington’s Disease-Associated Neurodegeneration. J. Clin. Investig..

[23] Alavi M.V., Fuhrmann N. (2013). Dominant Optic Atrophy, OPA1, and Mitochondrial Quality Control: Understanding Mitochondrial Network Dynamics. Mol. Neurodegener..

[24] Shirendeb U., Reddy A.P., Manczak M., Calkins M.J., Mao P., Tagle D.A., Reddy P.H. (2011). Abnormal Mitochondrial Dynamics, Mitochondrial Loss and Mutant Huntingtin Oligomers in Huntington’s Disease: Implications for Selective Neuronal Damage. Hum. Mol. Genet..

[25] Costa V., Giacomello M., Hudec R., Lopreiato R., Ermak G., Lim D., Malorni W., Davies K.J.A., Carafoli E., Scorrano L. (2010). Mitochondrial Fission and Cristae Disruption Increase the Response of Cell Models of Huntington’s Disease to Apoptotic Stimuli. EMBO Mol. Med..

[26] Kamitsuka P.J., Ghanem M.M., Ziar R., McDonald S.E., Thomas M.G., Kwakye G.F. (2023). Defective Mitochondrial Dynamics and Protein Degradation Pathways Underlie Cadmium-Induced Neurotoxicity and Cell Death in Huntington’s Disease Striatal Cells. Int. J. Mol. Sci..

[27] Beal M.F., Brouillet E., Jenkins B.G., Ferrante R.J., Kowall N.W., Miller J.M., Storey E., Srivastava R., Rosen B.R., Hyman B.T. (1993). Neurochemical and Histologic Characterization of Striatal Excitotoxic Lesions Produced by the Mitochondrial Toxin 3-Nitropropionic Acid. J. Neurosci..

[28] Túnez I., Tasset I., Pérez-De La Cruz V., Santamaría A. (2010). 3-Nitropropionic Acid as a Tool to Study the Mechanisms Involved in Huntington’s Disease: Past, Present and Future. Molecules.

[29] Browne S.E., Beal M.F. (2004). The Energetics of Huntington’s Disease. Neurochem. Res..

[30] Oz M., Lorke D.E., Hasan M., Petroianu G.A. (2011). Cellular and Molecular Actions of Methylene Blue in the Nervous System. Med. Res. Rev..

[31] Klosowski E.M., de Souza B.T.L., Mito M.S., Constantin R.P., Mantovanelli G.C., Mewes J.M., Bizerra P.F.V., Menezes P.V.M.d.C., Gilglioni E.H., Utsunomiya K.S. (2020). The Photodynamic and Direct Actions of Methylene Blue on Mitochondrial Energy Metabolism: A Balance of the Useful and Harmful Effects of This Photosensitizer. Free Radic. Biol. Med..

[32] Buchholz K., Schirmer R.H., Eubel J.K., Akoachere M.B., Dandekar T., Becker K., Gromer S. (2008). Interactions of Methylene Blue with Human Disulfide Reductases and Their Orthologues from Plasmodium Falciparum. Antimicrob. Agents Chemother..

[33] Bhurtel S., Bok E., Katila N., Kim J., Choi D.-Y. (2021). Activation of Nrf2 by Methylene Blue Is Associated with the Neuroprotection against MPP+ Induced Toxicity via Ameliorating Oxidative Stress and Mitochondrial Dysfunction. Biochem. Pharmacol..

[34] Gureev A.P., Shaforostova E.A., Popov V.N. (2019). Regulation of Mitochondrial Biogenesis as a Way for Active Longevity: Interaction Between the Nrf2 and PGC-1\alpha Signaling Pathways. Front. Genet..

[35] Gureev A.P., Sadovnikova I.S., Popov V.N. (2022). Molecular Mechanisms of the Neuroprotective Effect of Methylene Blue. Biochem. Biokhimiia.

[36] Medina D.X., Caccamo A., Oddo S. (2011). Methylene Blue Reduces A\beta Levels and Rescues Early Cognitive Deficit by Increasing Proteasome Activity. Brain Pathol..

[37] Gomez-Sequeda N., Jimenez-Del-Rio M., Velez-Pardo C. (2024). The Antiproteinopathy, Antioxidant, and Antiapoptotic Effects of Methylene Blue and 4-Phenylbutyric Acid Alone, and in Combination on Familial Alzheimer’s Disease PSEN1 I416T Cholinergic-Like Neurons. ACS Chem. Neurosci..

[38] Liu Y., Tan Y., Cheng G., Ni Y., Xie A., Zhu X., Yin C., Zhang Y., Chen T. (2024). Customized Intranasal Hydrogel Delivering Methylene Blue Ameliorates Cognitive Dysfunction against Alzheimer’s Disease. Adv. Mater..

[39] Rojas J.C., Simola N., Kermath B.A., Kane J.R., Schallert T., Gonzalez-Lima F. (2009). Striatal Neuroprotection with Methylene Blue. Neuroscience.

[40] Sontag E.M., Lotz G.P., Agrawal N., Tran A., Aron R., Yang G., Necula M., Lau A., Finkbeiner S., Glabe C. (2012). Methylene Blue Modulates Huntingtin Aggregation Intermediates and Is Protective in Huntington’s Disease Models. J. Neurosci..

[41] Gureev A.P., Shaforostova E.A., Laver D.A., Khorolskaya V.G., Syromyatnikov M.Y., Popov V.N. (2019). Methylene Blue Elicits Non-Genotoxic H2O2 Production and Protects Brain Mitochondria from Rotenone Toxicity. J. Appl. Biomed..

[42] Stack C., Jainuddin S., Elipenahli C., Gerges M., Starkova N., Starkov A.A., Jové M., Portero-Otin M., Launay N., Pujol A. (2014). Methylene Blue Upregulates Nrf2/ARE Genes and Prevents Tau-Related Neurotoxicity. Hum. Mol. Genet..

[43] Williams B.B., Li D., Wegrzynowicz M., Vadodaria B.K., Anderson J.G., Kwakye G.F., Aschner M., Erikson K.M., Bowman A.B. (2010). Disease-Toxicant Screen Reveals a Neuroprotective Interaction between Huntington’s Disease and Manganese Exposure. J. Neurochem..

[44] Dominah G.A., McMinimy R.A., Kallon S., Kwakye G.F. (2017). Acute Exposure to Chlorpyrifos Caused NADPH Oxidase Mediated Oxidative Stress and Neurotoxicity in a Striatal Cell Model of Huntington’s Disease. Neurotoxicology.

[45] Johri A., Calingasan N.Y., Hennessey T.M., Sharma A., Yang L., Wille E., Chandra A., Beal M.F. (2012). Pharmacologic Activation of Mitochondrial Biogenesis Exerts Widespread Beneficial Effects in a Transgenic Mouse Model of Huntington’s Disease. Hum. Mol. Genet..

[46] Chaturvedi R.K., Hennessey T., Johri A., Tiwari S.K., Mishra D., Agarwal S., Kim Y.S., Beal M.F. (2012). Transducer of Regulated CREB-Binding Proteins (TORCs) Transcription and Function Is Impaired in Huntington’s Disease. Hum. Mol. Genet..

[47] Zheng J., Winderickx J., Franssens V., Liu B. (2018). A Mitochondria-Associated Oxidative Stress Perspective on Huntington’s Disease. Front. Mol. Neurosci..

[48] Benchoua A., Trioulier Y., Zala D., Gaillard M.-C., Lefort N., Dufour N., Saudou F., Elalouf J.-M., Hirsch E., Hantraye P. (2006). Involvement of Mitochondrial Complex II Defects in Neuronal Death Produced by N-Terminus Fragment of Mutated Huntingtin. Mol. Biol. Cell.

[49] Goetzman E., Gong Z., Zhang B., Muzumdar R. (2023). Complex II Biology in Aging, Health, and Disease. Antioxidants.

[50] Bhurtel S., Katila N., Neupane S., Srivastav S., Park P.-H., Choi D.-Y. (2018). Methylene Blue Protects Dopaminergic Neurons against MPTP-Induced Neurotoxicity by Upregulating Brain-Derived Neurotrophic Factor. Ann. N. Y. Acad. Sci..

[51] Wen Y., Li W., Poteet E.C., Xie L., Tan C., Yan L.-J., Ju X., Liu R., Qian H., Marvin M.A. (2011). Alternative Mitochondrial Electron Transfer as a Novel Strategy for Neuroprotection. J. Biol. Chem..

[52] Liu S., Liu S., He B., Li L., Li L., Wang J., Cai T., Chen S., Jiang H. (2021). OXPHOS Deficiency Activates Global Adaptation Pathways to Maintain Mitochondrial Membrane Potential. EMBO Rep..

[53] Kensler T.W., Wakabayashi N., Biswal S. (2007). Cell Survival Responses to Environmental Stresses via the Keap1-Nrf2-ARE Pathway. Annu. Rev. Pharmacol. Toxicol..

[54] Ighodaro O.M., Akinloye O.A. (2018). First Line Defence Antioxidants-Superoxide Dismutase (SOD), Catalase (CAT) and Glutathione Peroxidase (GPX): Their Fundamental Role in the Entire Antioxidant Defence Grid. Alex. J. Med..

[55] van Roon-Mom W.M.C., Pepers B.A., ’t Hoen P.A.C., Verwijmeren C.A.C.M., den Dunnen J.T., Dorsman J.C., van Ommen G.B. (2008). Mutant Huntingtin Activates Nrf2-Responsive Genes and Impairs Dopamine Synthesis in a PC12 Model of Huntington’s Disease. BMC Mol. Biol..

[56] Lee K.K., Boelsterli U.A. (2014). Bypassing the Compromised Mitochondrial Electron Transport with Methylene Blue Alleviates Efavirenz/Isoniazid-Induced Oxidant Stress and Mitochondria-Mediated Cell Death in Mouse Hepatocytes. Redox Biol..

[57] Weydt P., Pineda V.V., Torrence A.E., Libby R.T., Satterfield T.F., Lazarowski E.R., Gilbert M.L., Morton G.J., Bammler T.K., Strand A.D. (2006). Thermoregulatory and Metabolic Defects in Huntington’s Disease Transgenic Mice Implicate PGC-1alpha in Huntington’s Disease Neurodegeneration. Cell Metab..

[58] Bhatti J.S., Bhatti G.K., Reddy P.H. (2017). Mitochondrial Dysfunction and Oxidative Stress in Metabolic Disorders—A Step towards Mitochondria Based Therapeutic Strategies. Biochim. Biophys. Acta Mol. Basis Dis..

[59] Wu S., Zhou F., Zhang Z., Xing D. (2011). Mitochondrial Oxidative Stress Causes Mitochondrial Fragmentation via Differential Modulation of Mitochondrial Fission-Fusion Proteins. FEBS J..

[60] Joshi A.U., Ebert A.E., Haileselassie B., Mochly-Rosen D. (2019). Drp1/Fis1-Mediated Mitochondrial Fragmentation Leads to Lysosomal Dysfunction in Cardiac Models of Huntington’s Disease. J. Mol. Cell. Cardiol..

[61] Filomeni G., De Zio D., Cecconi F. (2015). Oxidative Stress and Autophagy: The Clash between Damage and Metabolic Needs. Cell Death Differ..

[62] Trettel F., Rigamonti D., Hilditch-Maguire P., Wheeler V.C., Sharp A.H., Persichetti F., Cattaneo E., MacDonald M.E. (2000). Dominant Phenotypes Produced by the HD Mutation in STHdh(Q111) Striatal Cells. Hum. Mol. Genet..

