# Supplementary material for: Methylene Blue Attenuates 3-Nitropropionic Acid-Induced Oxidative Stress and Mitochondrial Dysfunction in Striatal Cells: Therapeutic Implications in Huntington’s Disease Neuropathology
Source: Int J Mol Sci. 2025 Nov 1;26(21):10672. doi: 10.3390/ijms262110672 (PMC12610986; doi:10.3390/ijms262110672)

NRF2 (Set F)

Loading order - WT 0, WT 3NPA, WT MB, WT 3NPA + MB, HD 0, HD 3NPA, HD MB, HD 3 NPA+ MB

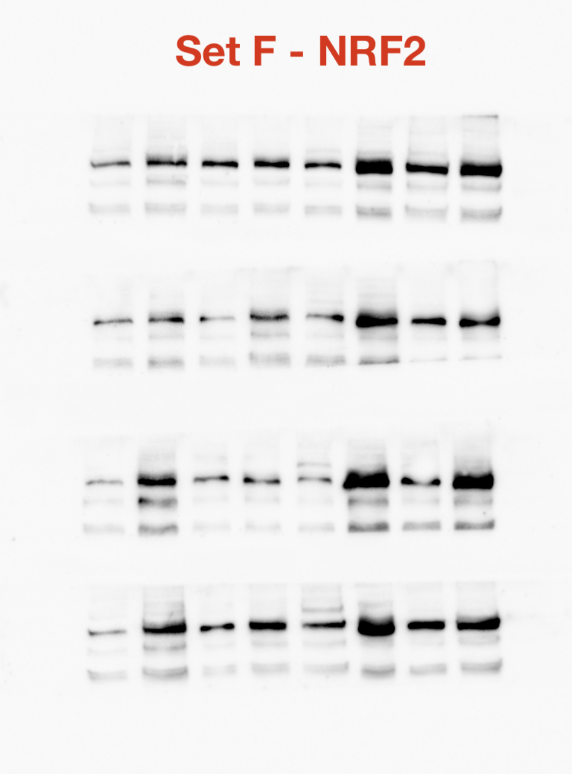

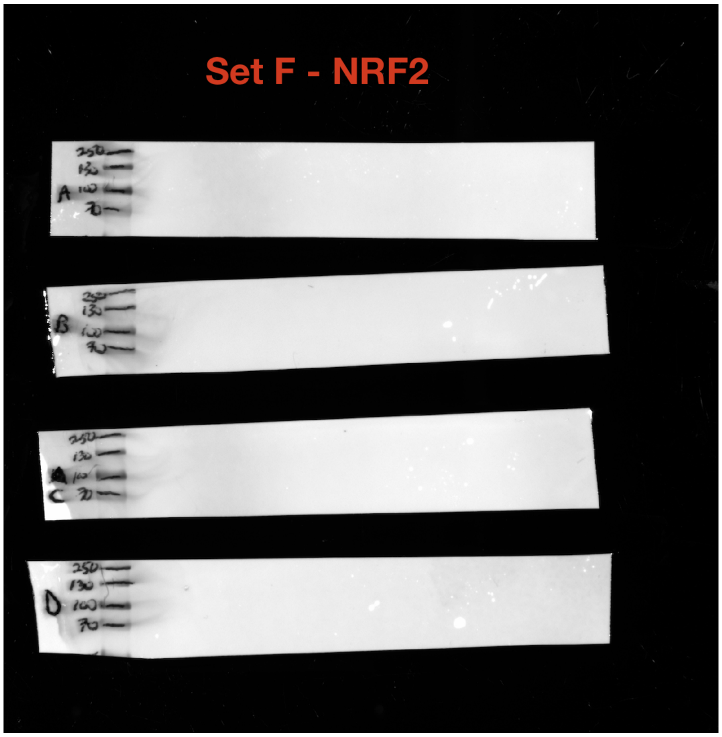

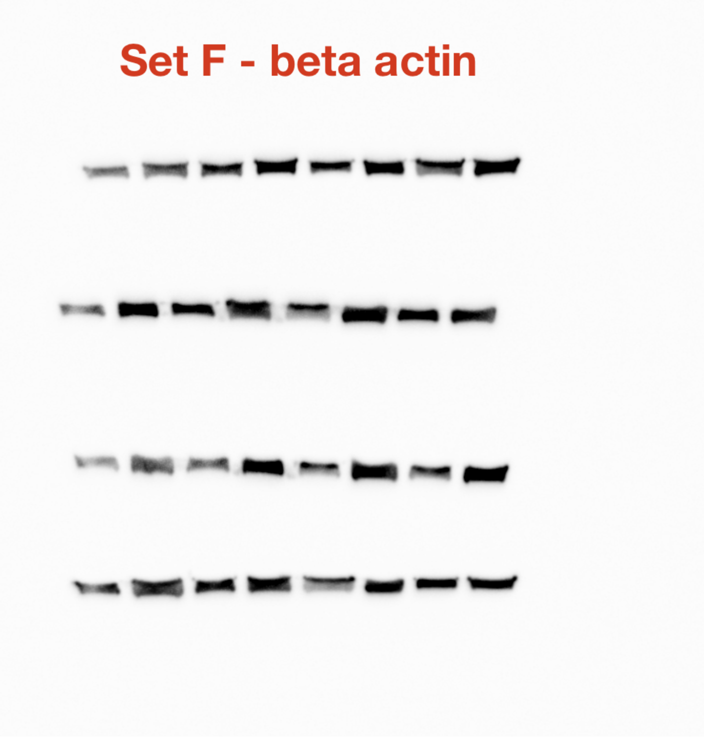

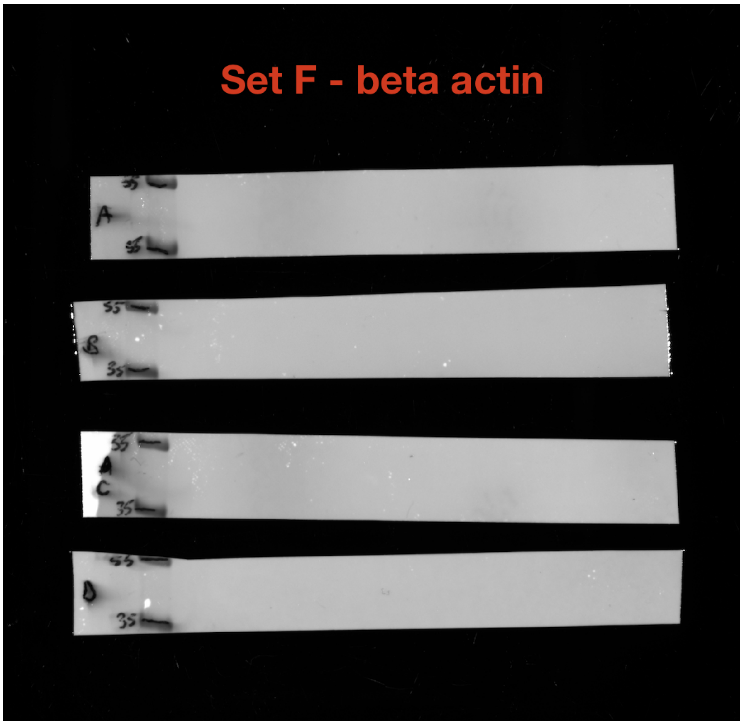

HO1 (Set B)

Loading order - WT 0, WT 3NPA, WT 3 NPA + MB, WT MB, HD 0, HD 3NPA, HD 3 NPA + MB, HD MB

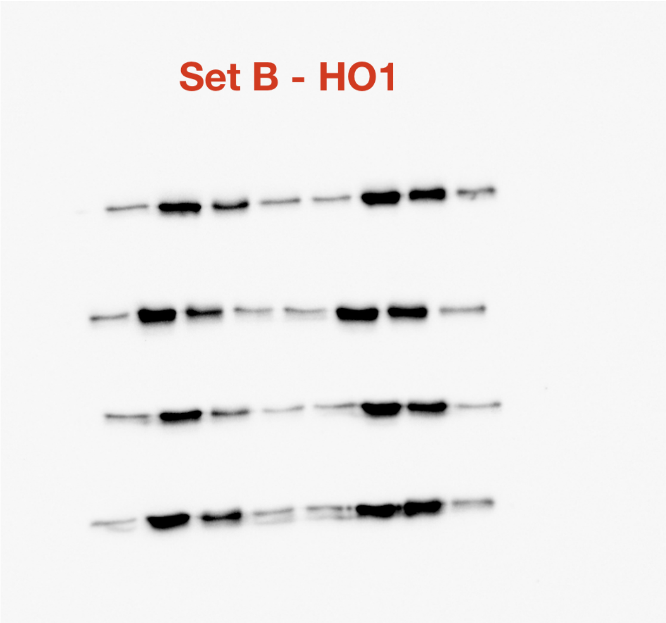

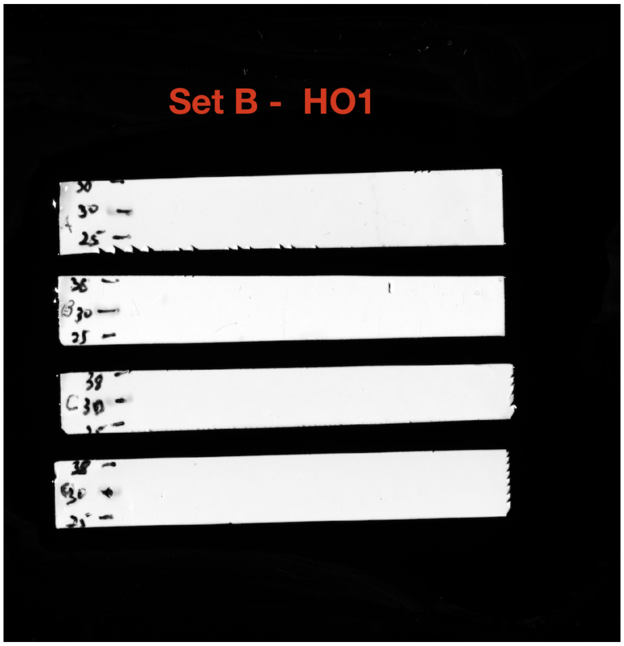

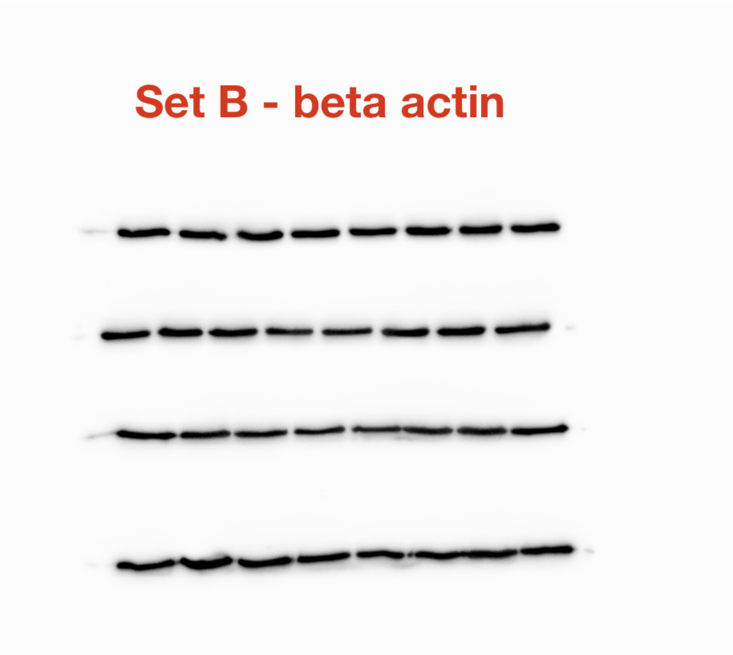

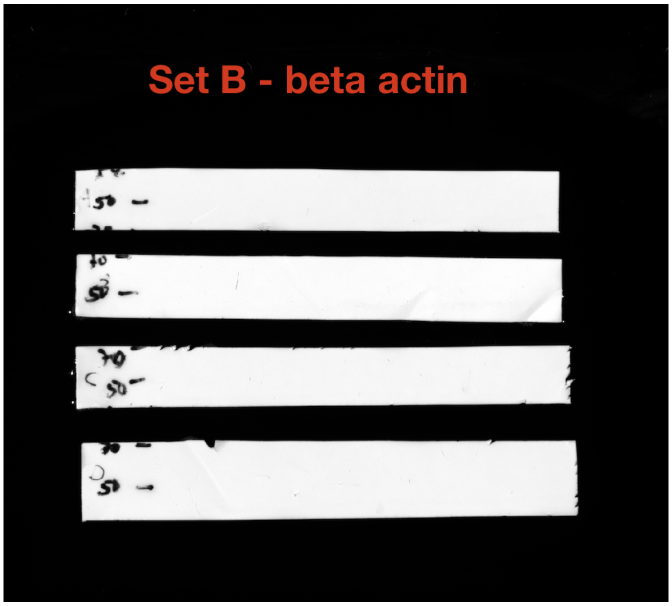

SOD1 - SET B

Loading order - WT 0, WT 3NPA, WT 3 NPA + MB, WT MB, HD 0, HD 3NPA, HD 3 NPA + MB, HD MB

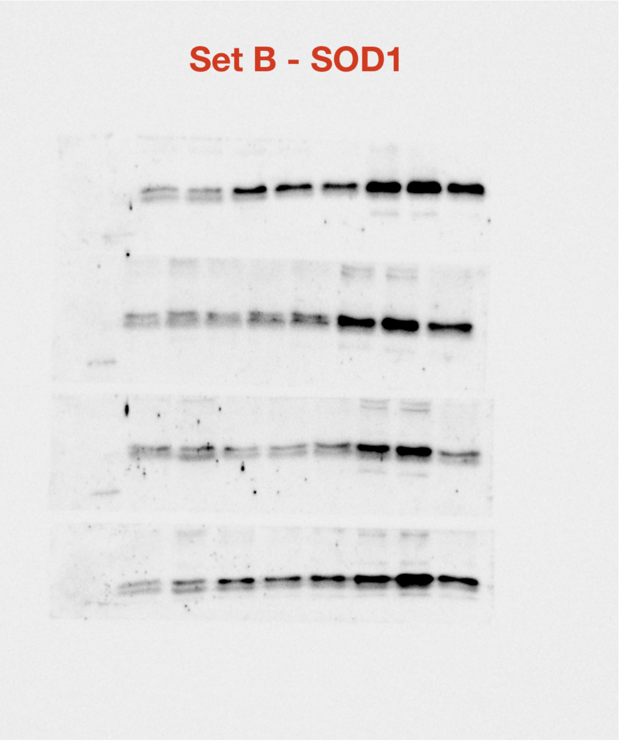
Set B - SOD1

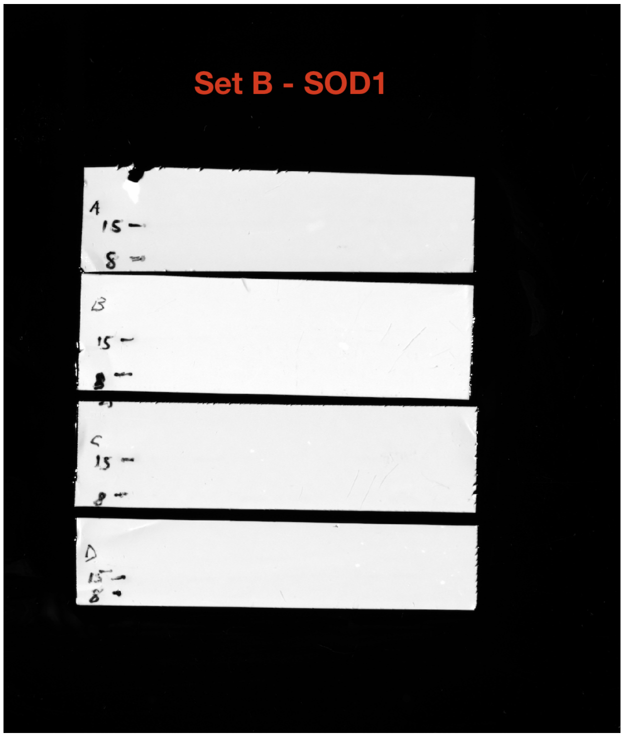
Set B - SOD1
A
15
8
B
15
8
C
15
8
D
15
8

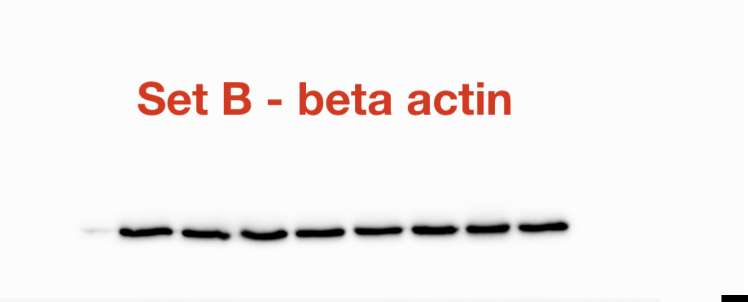

SOD2 (Set F)

Loading order - WT 0, WT 3NPA, WT MB, WT 3NPA + MB, HD 0, HD 3NPA, HD MB, HD 3 NPA+ MB

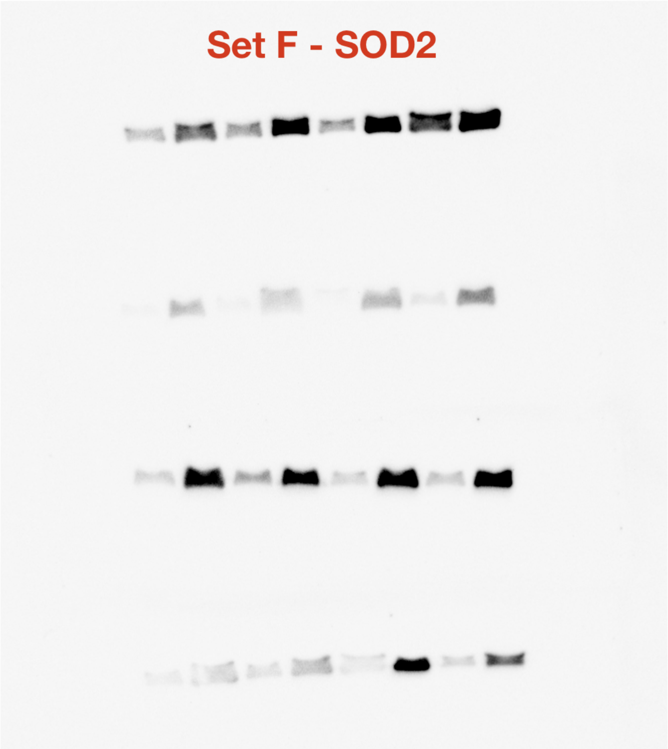

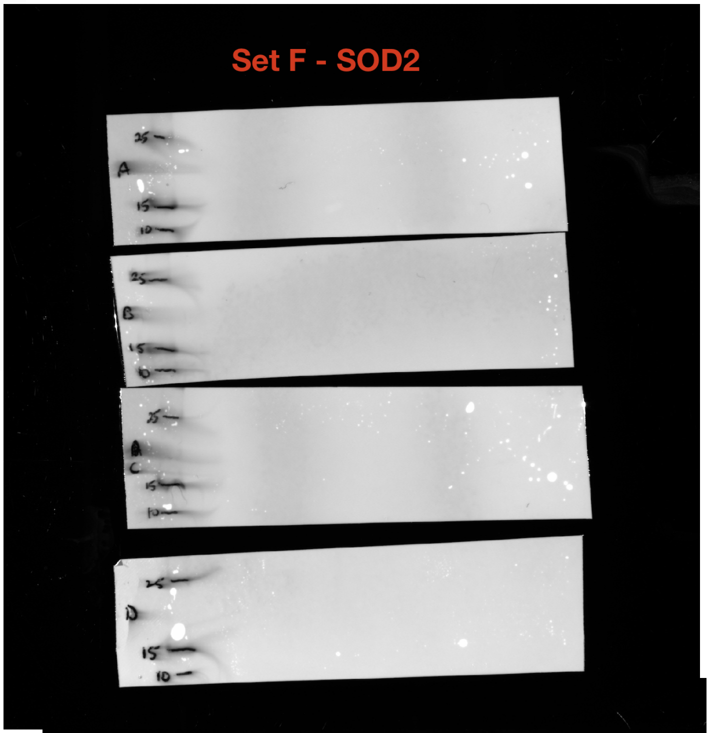

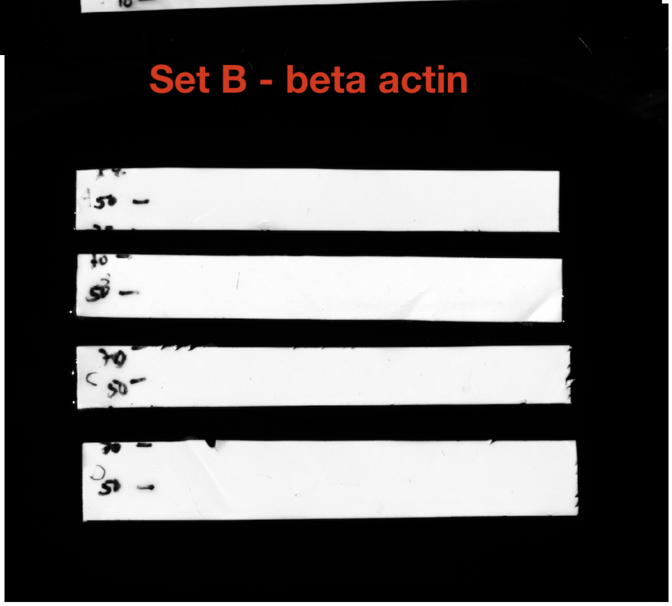

PGC1-a (Set 2)

Loading order - WT 0, WT 3NPA, WT 3 NPA + MB, WT MB, HD 0, HD 3NPA, HD 3 NPA + MB, HD MB

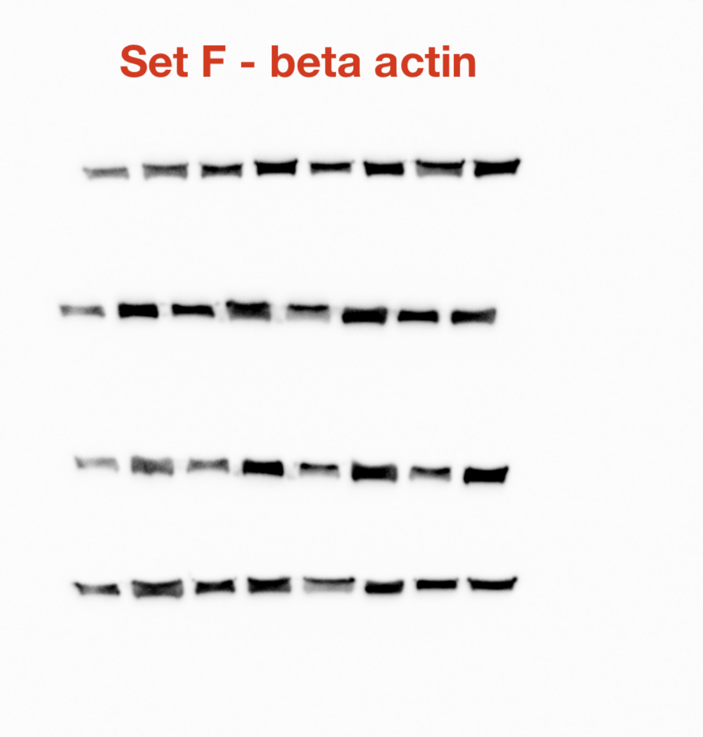

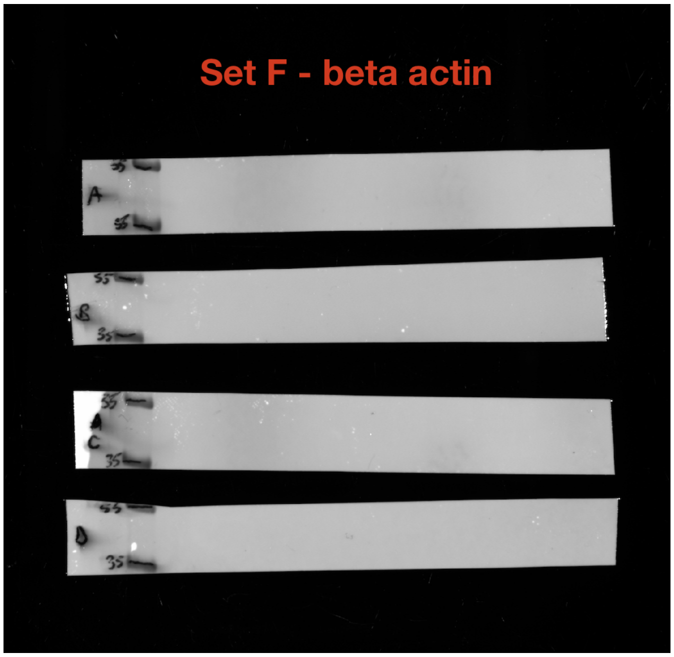

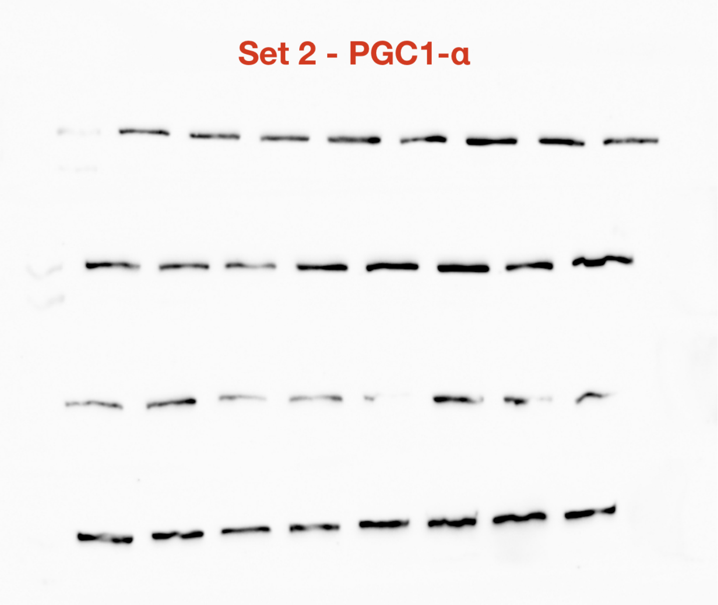

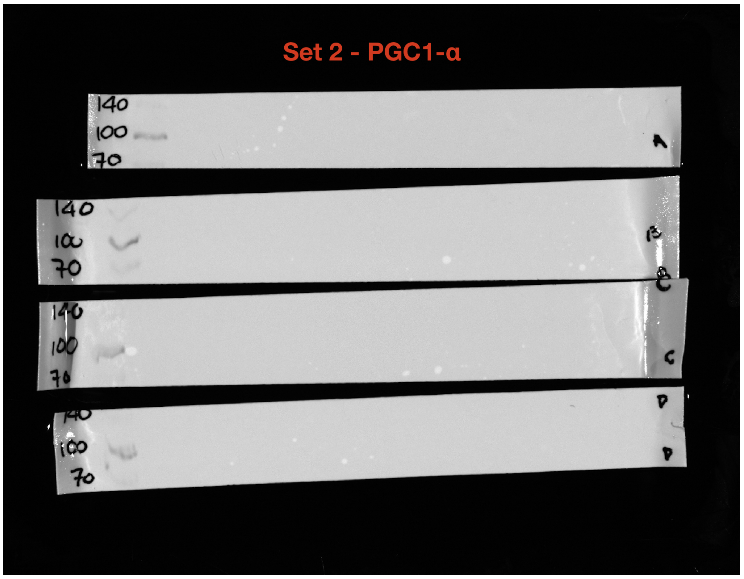

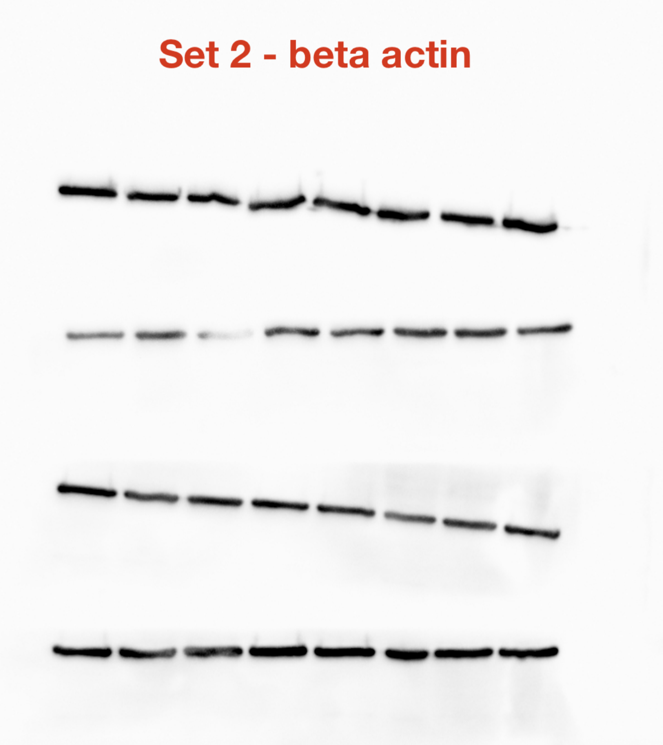

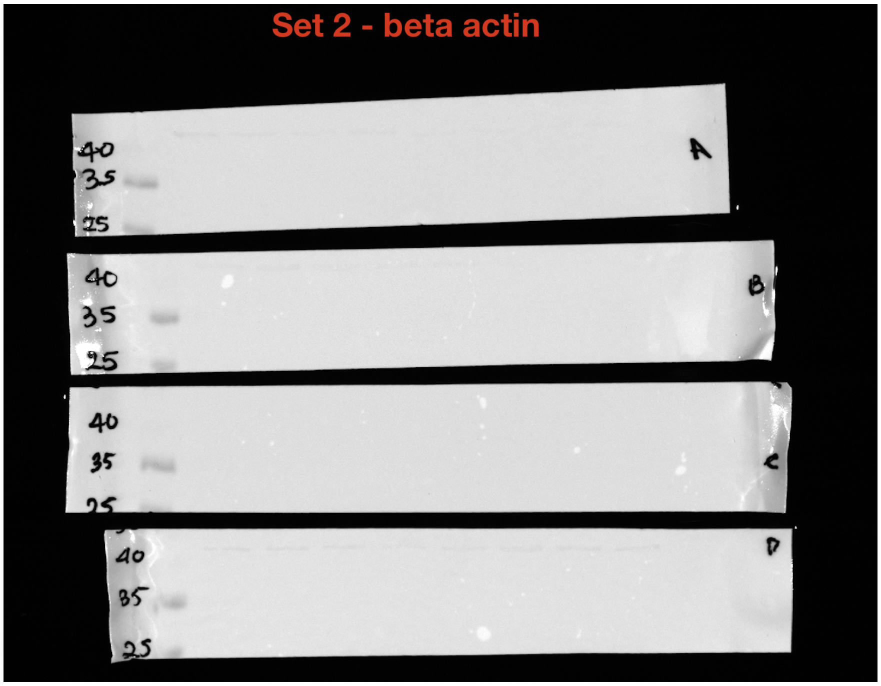

Fis1 (Set B)

Loading order - WT 0, WT 3NPA, WT 3 NPA + MB, WT MB, HD 0, HD 3NPA, HD 3 NPA + MB, HD MB

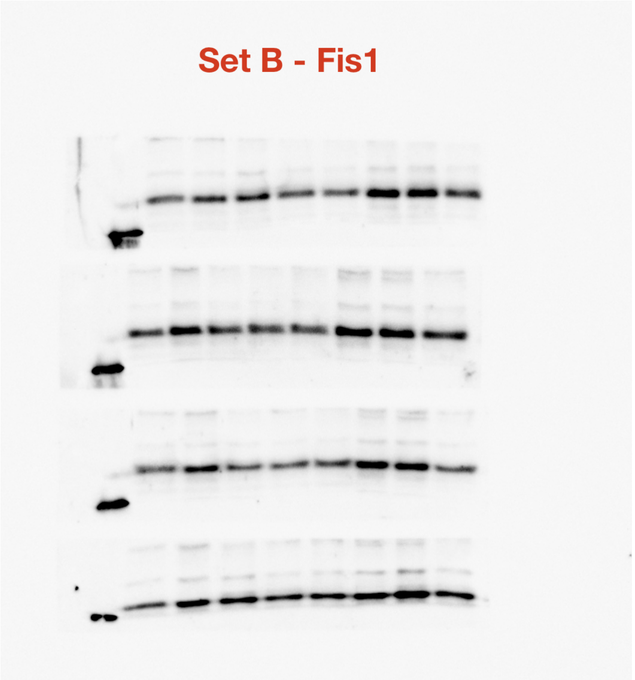
Set B - Fis1

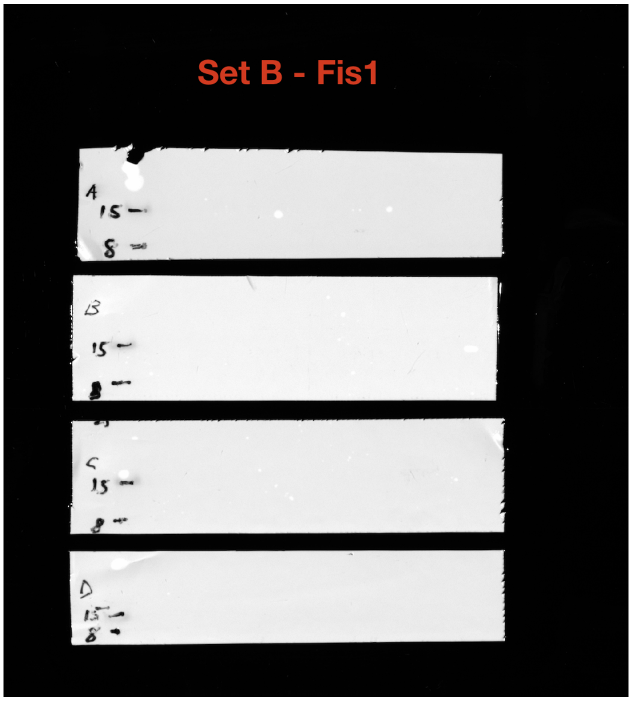
Set B - Fis1
A
15
8
B
15
8
C
15
8
D
15
8

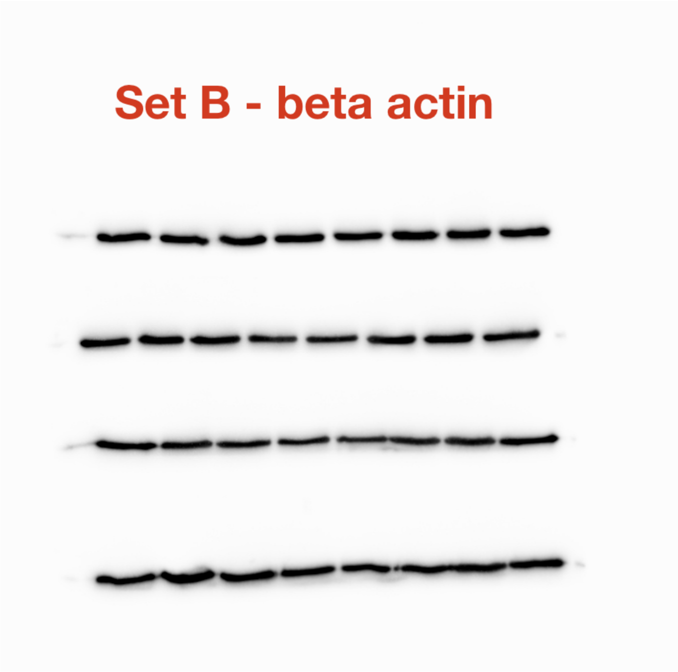

DRP1 (Set E)

Loading order WT 0, WT 3NPA, WT 3 NPA + MB, WT MB, HD 0, HD 3NPA, HD 3 NPA + MB, HD MB

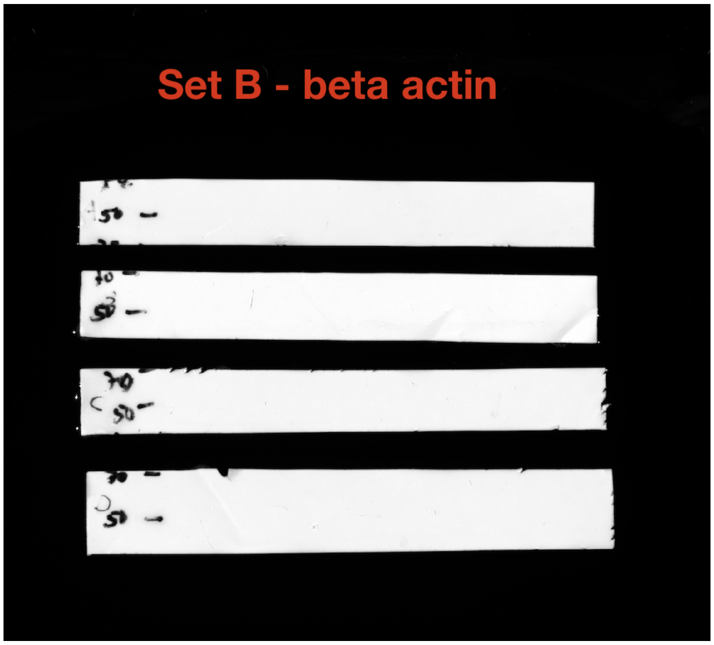

Set E - DRP1

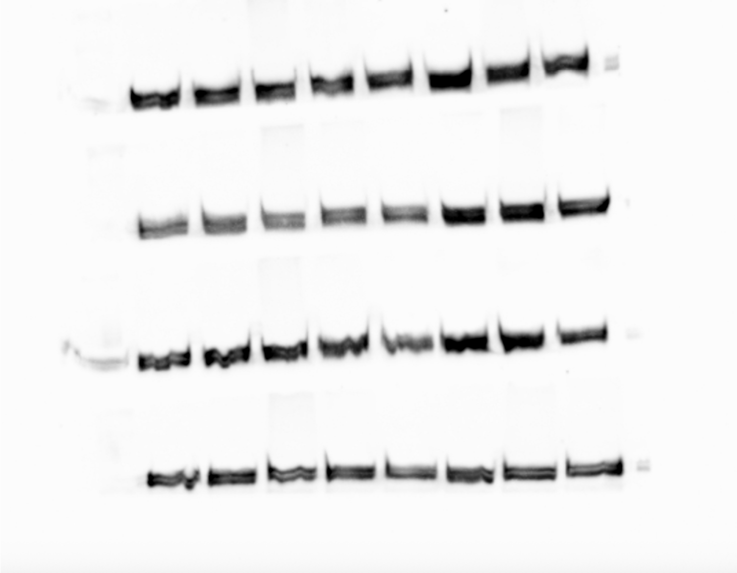

Set E - DRP1

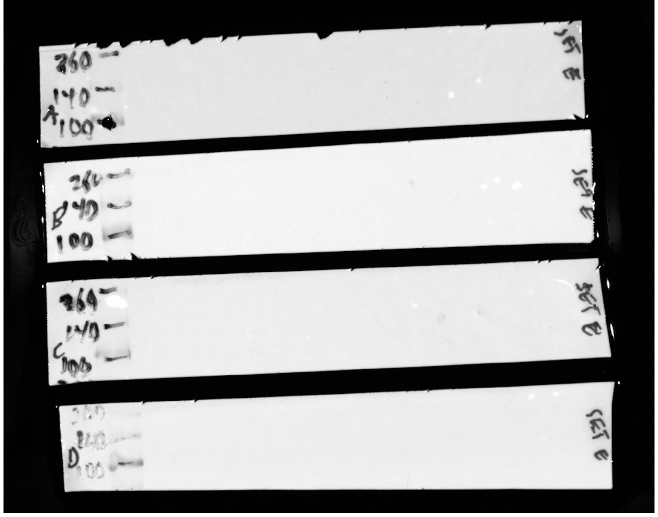

Set E - beta actin

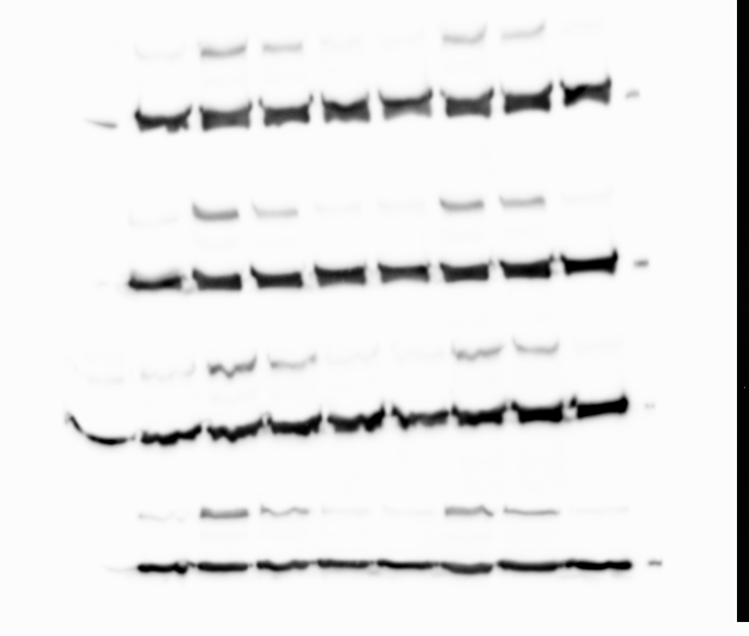

Set E - beta actin

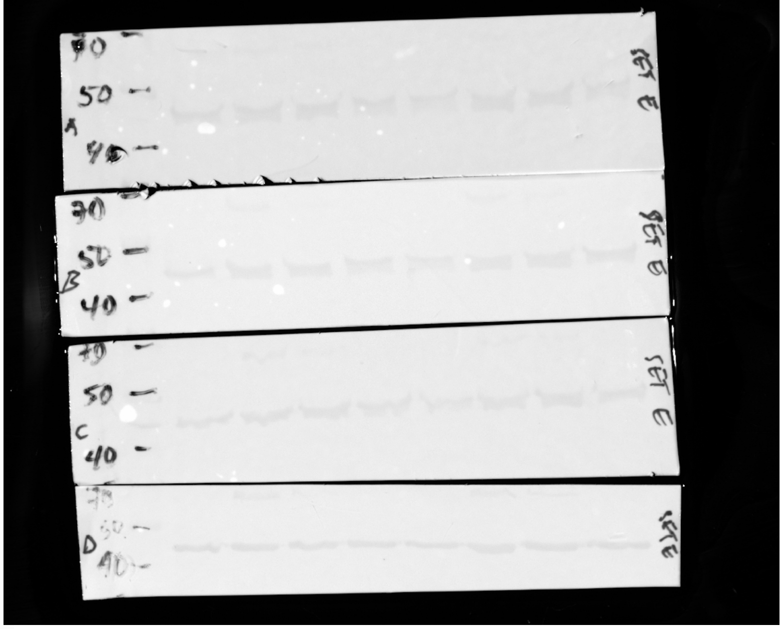

OPA1 (Set D)

Loading order WT 0, WT 3NPA, WT 3 NPA + MB, WT MB, HD 0, HD 3NPA, HD 3 NPA + MB, HD MB

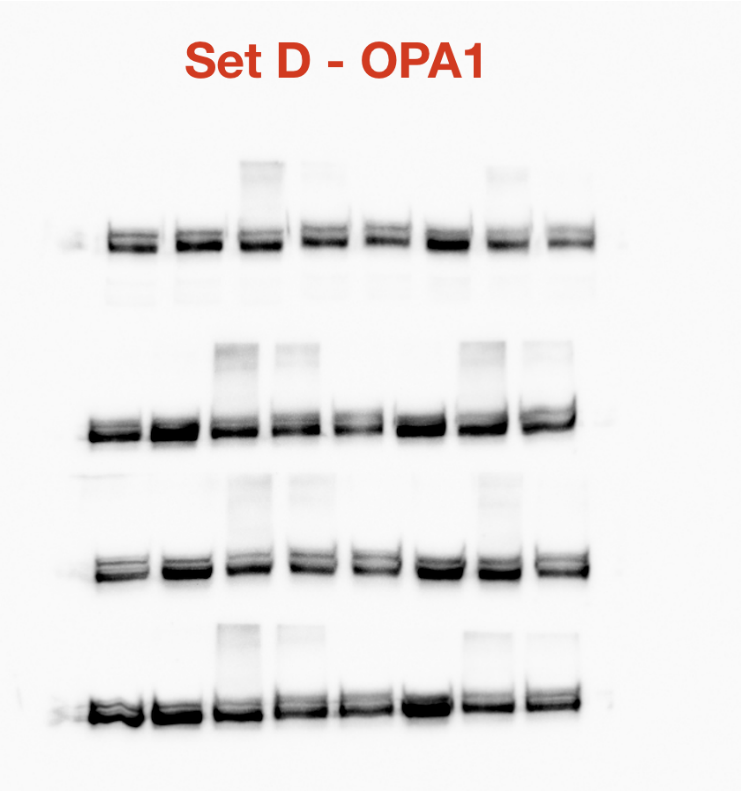

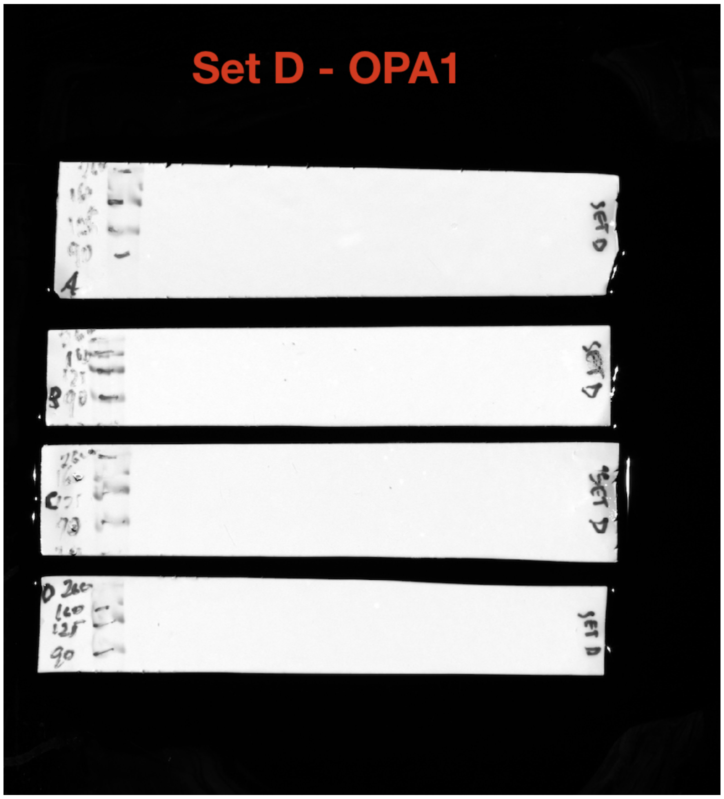

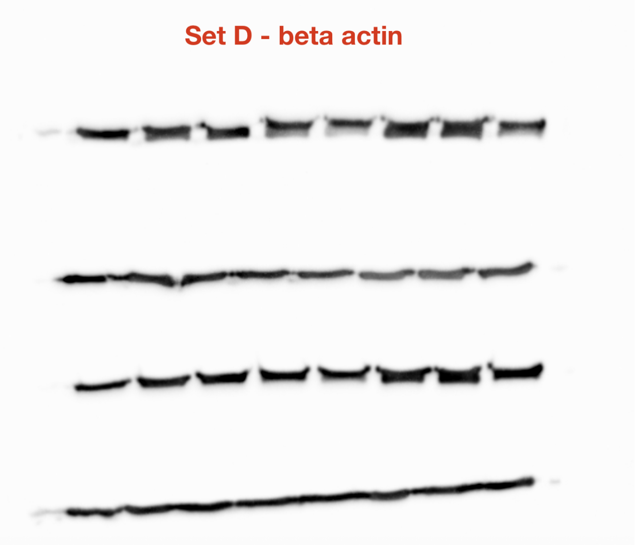

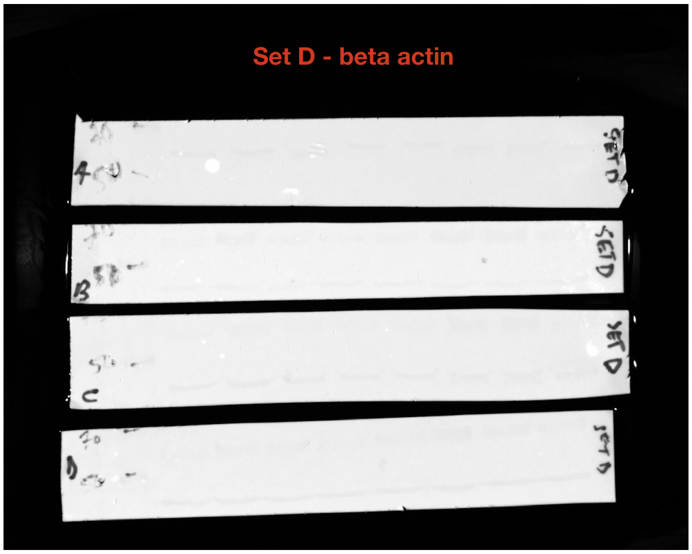

KEAP1

Loading order WT 0, WT 3NPA, WT 3 NPA + MB, WT MB, HD 0, HD 3NPA, HD 3 NPA + MB, HD MB

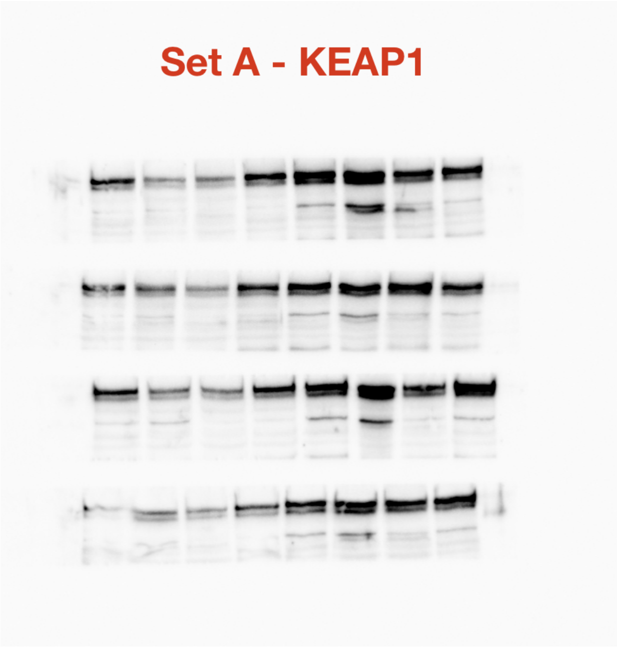
Set A - KEAP1

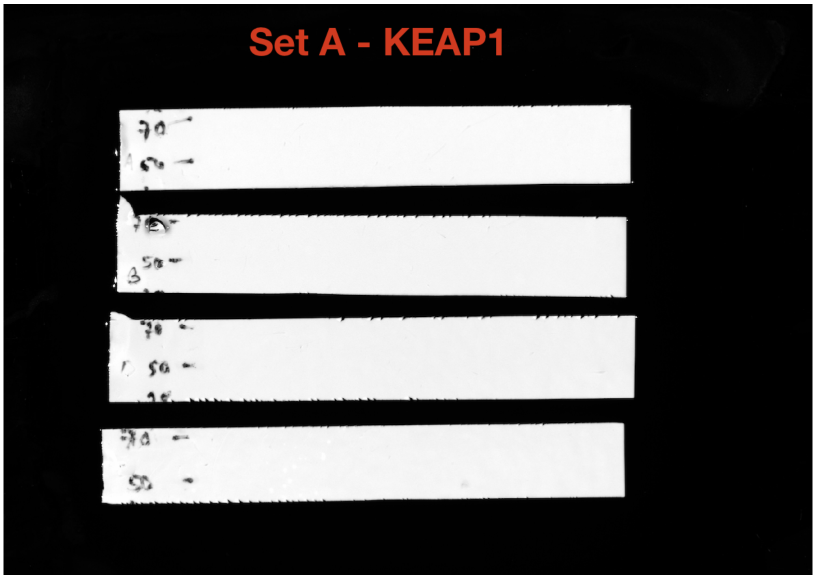
Set A - KEAP1

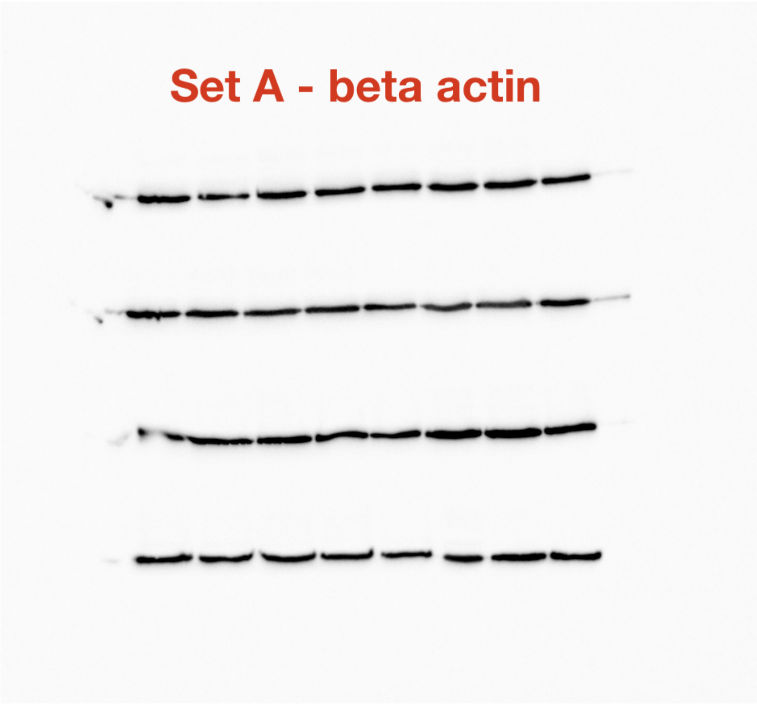

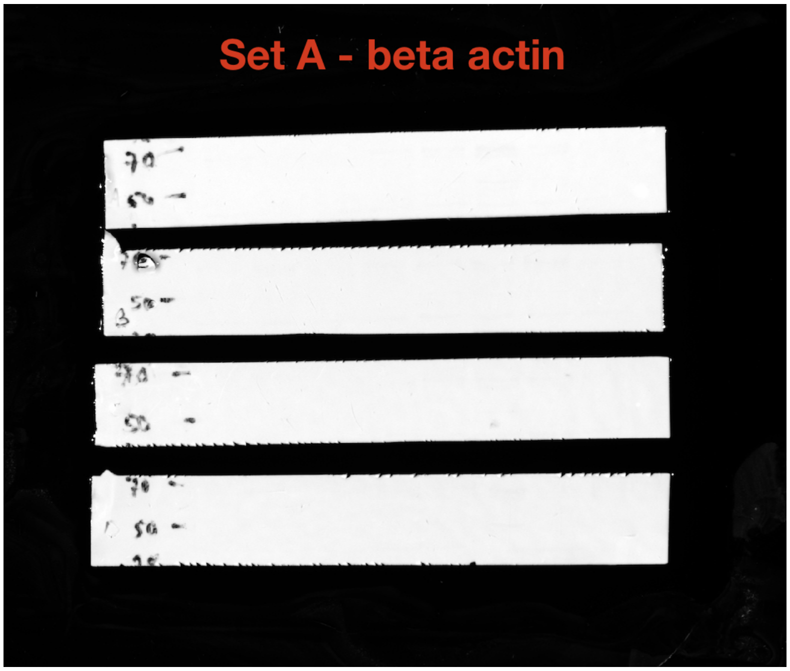

P62 - Set D

Loading order - WT 0, WT 3NPA, WT 3 NPA + MB, WT MB, HD 0, HD 3NPA, HD 3 NPA + MB, HD MB

Set E - p62

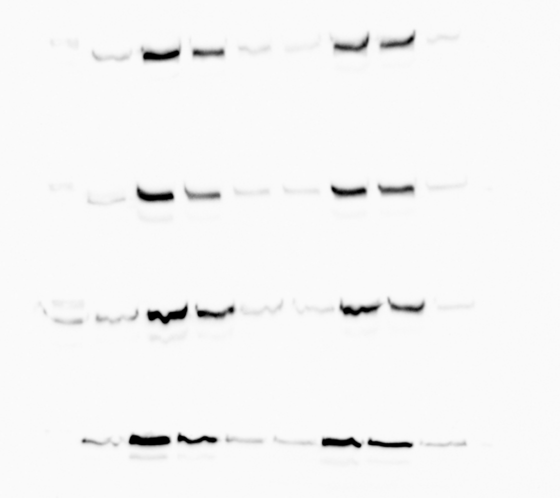

Set E - p62

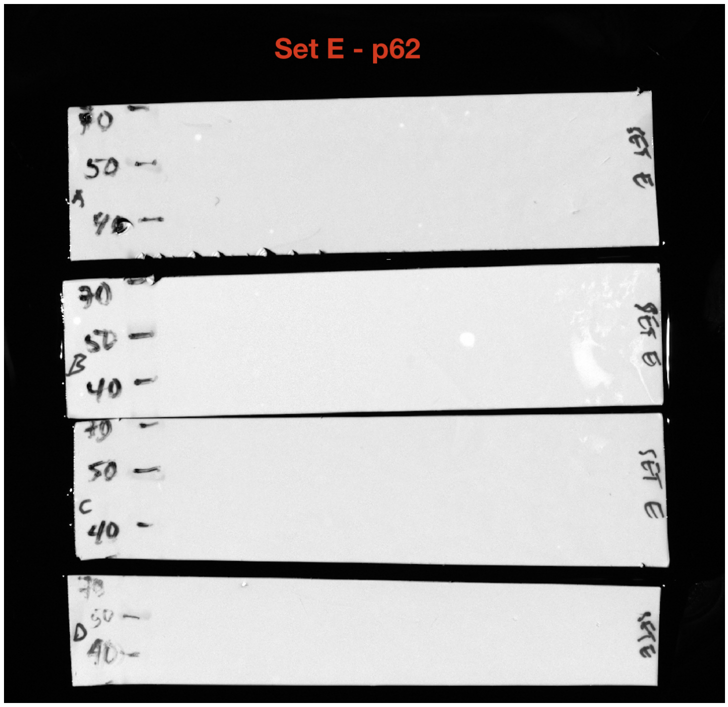

Set E - beta actin

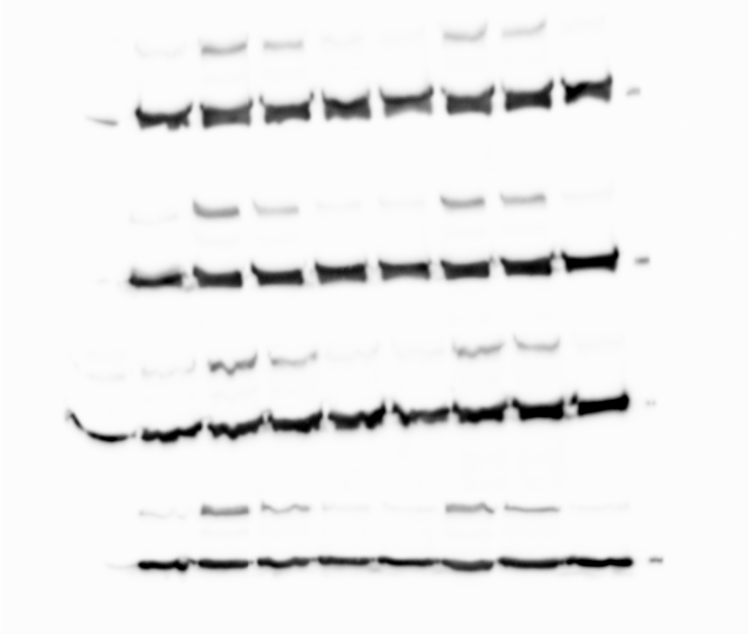

Set E - beta actin

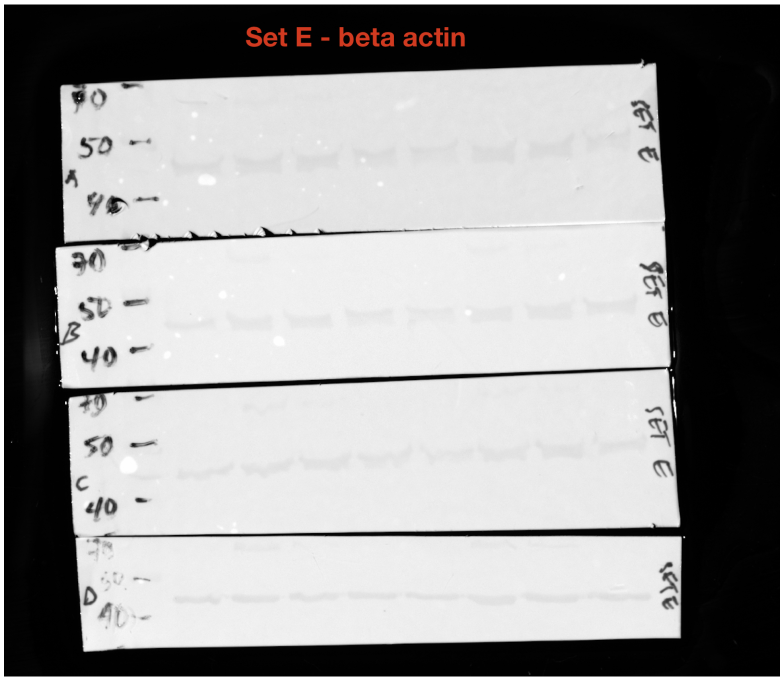

MFN2 - Set 3

Loading order (7 WT conditions, 7 HD conditions)

WT

0   + H2O2   + MB   + MB & 3NPA   + MB & Rotenont   +3-NPA   + Rotenone

HD

0   + H2O2   + MB   + MB & 3NPA   + MB & Rotenont   +3-NPA   + Rotenone

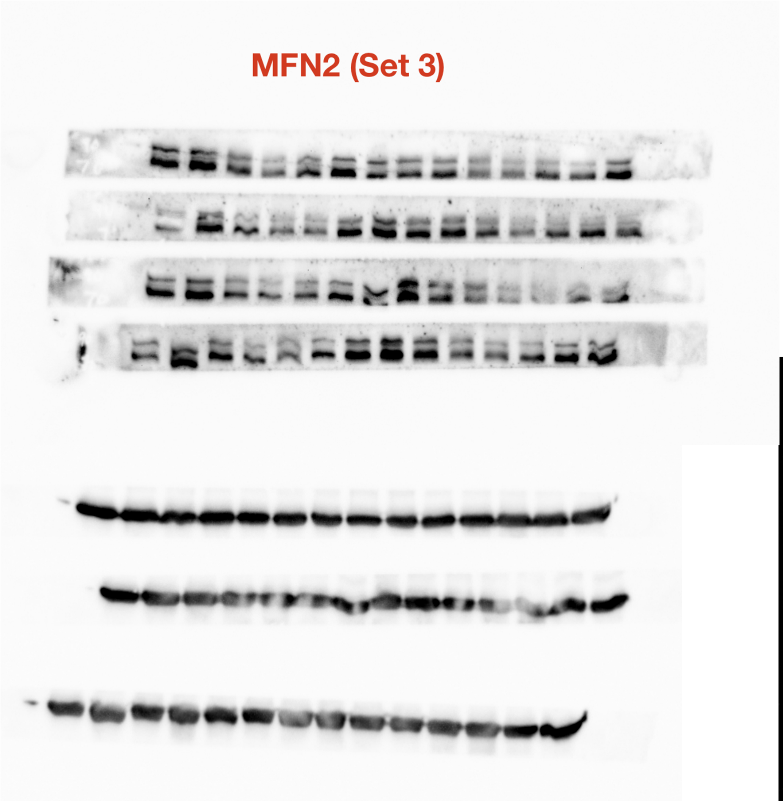

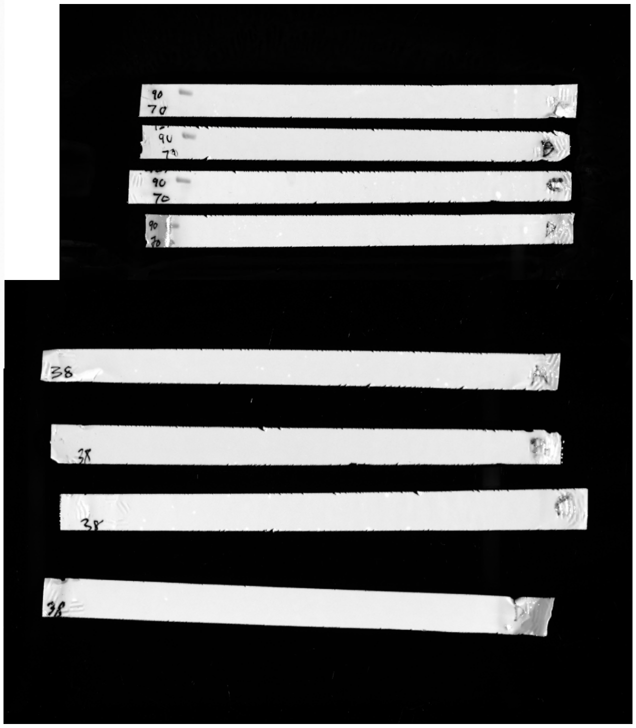

p-DRP1 - Set 3

Loading order (7 WT conditions, 7 HD conditions)

WT

0 + H2O2 + MB + MB & 3NPA + MB & Rotenone +3-NPA + Rotenone

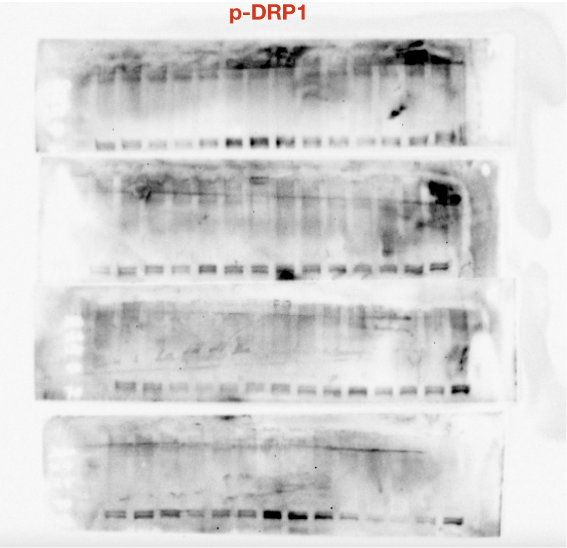

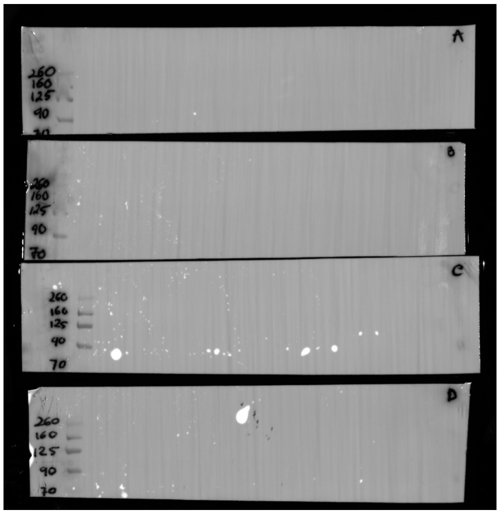

HD

0 + H2O2 + MB + MB & 3NPA + MB & Rotenone +3-NPA + Rotenone

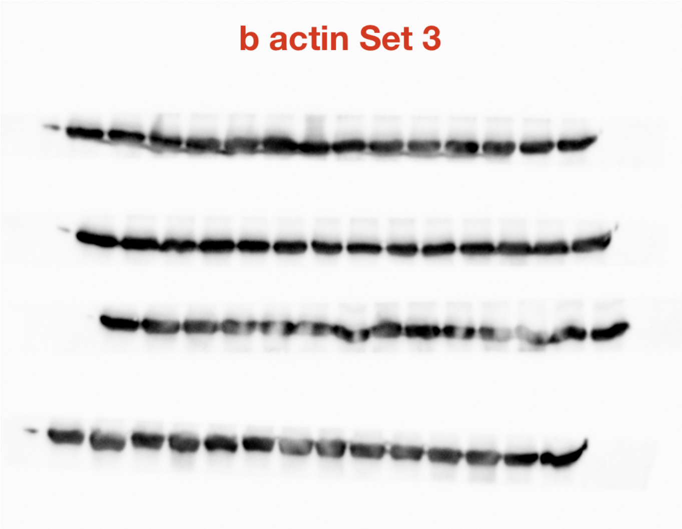

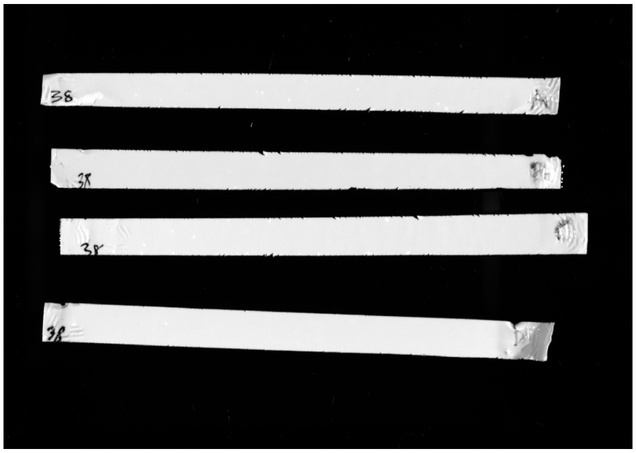

DRP1 - Set 4 (same cells as Set 3)

Loading order (7 WT conditions, 7 HD conditions)

WT

0 + H2O2 + MB + MB & 3NPA + MB & Rotenone +3-NPA + Rotenone

HD

0 + H2O2 + MB + MB & 3NPA + MB & Rotenone +3-NPA + Rotenone

DRP1

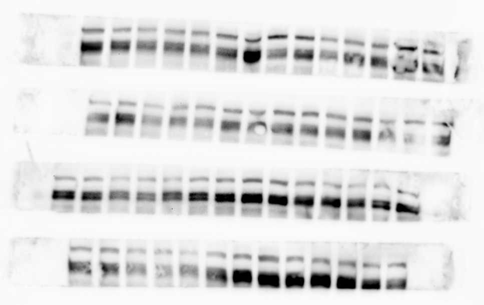

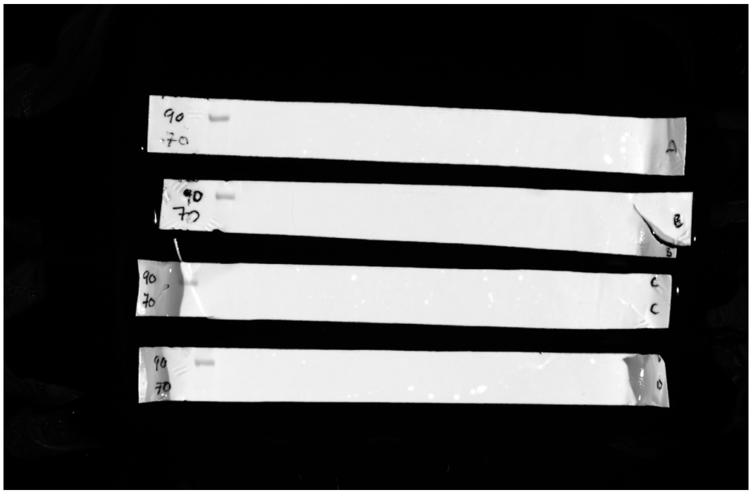

b actin Set 4

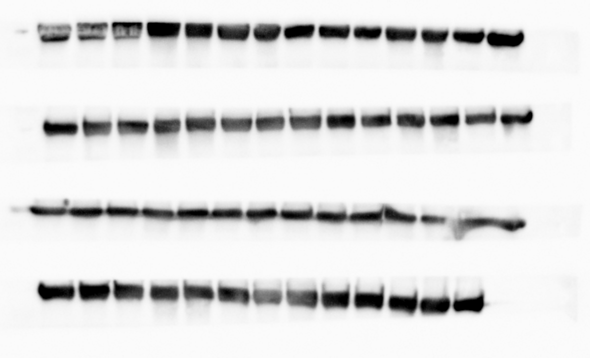

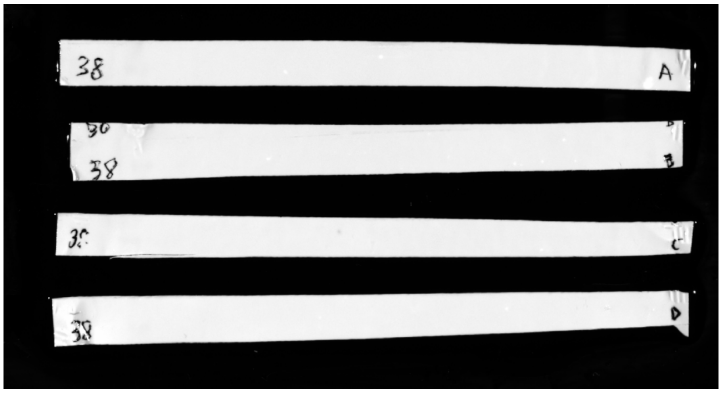

Supplement: Supplementary file 1 [file ijms-26-10672-s001.zip › ijms-3888769-supplementary.pdf]
